# The Time-resolved and Extreme-conditions XAS (TEXAS) facility at the European Synchrotron Radiation Facility: the energy-dispersive X-ray absorption spectroscopy beamline ID24

**DOI:** 10.1107/S160057751501783X

**Published:** 2016-01-01

**Authors:** S. Pascarelli, O. Mathon, T. Mairs, I. Kantor, G. Agostini, C. Strohm, S. Pasternak, F. Perrin, G. Berruyer, P. Chappelet, C. Clavel, M. C. Dominguez

**Affiliations:** a European Synchrotron Radiation Facility, 71 Avenue des Martyrs, 38000 Grenoble, France; b Deutsches Elektronen Synchrotron DESY, Notkestrasse 85, 22607 Hamburg, Germany

**Keywords:** X-ray absorption spectroscopy, XAS, energy-dispersive XAS, time-resolved XAS, XAS at extreme conditions

## Abstract

The European Synchrotron Radiation Facility has recently made available to the user community a facility totally dedicated to Time-resolved and Extreme-conditions X-ray Absorption Spectroscopy – TEXAS. Based on an upgrade of the former energy-dispersive XAS beamline ID24, it provides a unique experimental tool combining unprecedented brilliance (up to 10^14^ photons s^−1^ on a 4 µm × 4 µm FWHM spot) and detection speed for a full EXAFS spectrum (100 ps per spectrum). The science mission includes studies of processes down to the nanosecond timescale, and investigations of matter at extreme pressure (500 GPa), temperature (10000 K) and magnetic field (30 T). The core activities of the beamline are centered on new experiments dedicated to the investigation of extreme states of matter that can be maintained only for very short periods of time. Here the infrastructure, optical scheme, detection systems and sample environments used to enable the mission-critical performance are described, and examples of first results on the investigation of the electronic and local structure in melts at pressure and temperature conditions relevant to the Earth’s interior and in laser-shocked matter are given.

## Abstract 2

The new energy-dispersive XAS beamline at the European Synchrotron Radiation Facility is presented. A technical description of the beamline (optical scheme, detection, sample environments) is provided and its performance is illustrated with a few recent examples of experiments by different user groups.

## 1. Introduction   

The TEXAS facility is part of an ambitious project spread over more than ten years to upgrade the ESRF accelerator, beamlines and infrastructure. The Time-resolved and Extreme-conditions X-ray Absorption Spectroscopy (TEXAS) beamline is based on an upgrade of the former energy-dispersive X-ray absorption spectroscopy (EDXAS) beamline ID24. TEXAS offers the user community new opportunities for investigating rapid processes down to the nanosecond timescale, and matter at extreme conditions of pressure, temperature and magnetic field.

**Figure 1:**
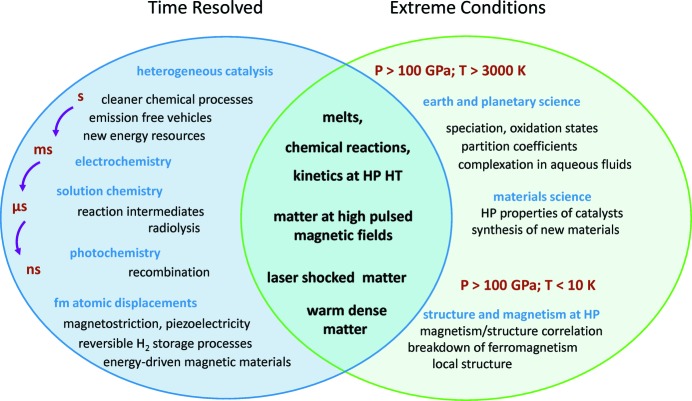
Scientific case for TEXAS: the core of the future activities lies in the intersection area between the ‘time-resolved’ and the ‘extreme conditions’ ellipses.

The scientific case for TEXAS is schematically illustrated in Fig. 1 ▸. The core of the future activities lies at the intersection between ‘time resolved’ and ‘extreme conditions’ applications. Whereas XAS at the ESRF and elsewhere addressed applications requiring rapid data acquisition or extreme environments, applications requiring both are still largely unexplored. The underlying reason is the stringent time resolution (below the millisecond in most cases) coupled to small focal spot (few micrometers) that is difficult to achieve simultaneously. The main motivation for the upgrade of ID24 was to make these new experiments possible.

Fast measurements of extreme states of matter is an increasingly important area of science. The fundamental importance of understanding the properties of matter at extreme conditions of pressure, temperature and magnetic field stems from the fact that ‘ambient’ conditions, those we experience at the surface of our planet, are not at all ‘ordinary’. In our universe, most condensed matter is found inside planets at pressures of several hundreds of GigaPascals (GPa) and thousands of Kelvin, while most atomic and molecular matter is found in stars, where magnetic fields can reach millions of Tesla. Such extreme conditions can modify chemical bonds and induce myriad changes in materials. The literature of the past few decades is full of pressure-induced electronic, magnetic and structural phase transitions: insulator-to-metal transitions, breakdown of ferromagnetism, onset of superconductivity, valence crossovers, triggering of chemical reactions, *etc*. (Minomura & Drickamer, 1962 ▸; Bastea *et al.*, 2001 ▸; Citroni *et al.*, 2002 ▸; Torchio *et al.*, 2011 ▸; Gavriliuk *et al.*, 2012 ▸). Reports of high magnetic field induced effects include new spin structures of anti-ferromagnetically coupled sublattices, magnetic quantum phase transitions, and various exotic phenomena such as non-Fermi liquid, unconventional superconductivity *etc*. (Wu *et al.*, 2013 ▸; Shekhter *et al.*, 2013 ▸). Achieving such conditions in the laboratory while probing structure and electronic and magnetic properties is important not only from a fundamental point of view or to understand our solar system and beyond but also for the synthesis and design of new materials with advanced properties. Since many of the physical and chemical consequences of extreme pressure, temperature and magnetic fields, such as changes in local structure, melting, modifications of the valence state, breaking or formation of chemical bonds, changes in sublattice magnetizations, are optimally observed through XAS, and since time resolution is key to the achievement of such extreme conditions, a high-brilliance EDXAS beamline is an ideal playground for such investigations.

One of the motivations that led to the TEXAS upgrade was the reduction in focal spot size, which opened a number of new opportunities for investigating matter at extreme conditions of pressure, temperature and magnetic field. For example, conditions similar to Earth’s deep interior at the relevant pressure and temperature conditions of the lower mantle had become accessible (Boehler *et al.*, 2009 ▸; Andrault *et al.*, 2010 ▸; Narygina *et al.*, 2011 ▸). *In situ* hyperspectral mapping, with spatial resolution of a few micrometers, of samples in a diamond anvil cell also revealed new diagnostics methods for quantification of thermal and pressure gradients (Muñoz *et al.*, 2008 ▸; Aquilanti *et al.*, 2009*a* ▸). The availability of element-selective, chemical and structural information in a time-resolved mode opened the exciting possibility of following chemical reactions, or decompositions, at extreme conditions, in real time. But, the full exploitation of a 4 µm × 4 µm spot required a new beamline design based on new stability and precision standards.

Also, the reduction in spot size penalized important applications that required spatial average on samples with heterogeneities on the scale of tens of micrometers; the investigation of heterogeneous catalysts *in operando* is an example (Bordiga *et al.*, 2013 ▸). The scientific impact of these studies is immediate as they concern preservation of the environment, air and water pollution, the search for new cleaner energy resources, sustainable development, *etc*. They involve time-resolved *in-situ* XAS investigations of supported metal nanoparticles used as active catalysts for the automotive industry, or of toxic metals in soils and water, or catalysts involved in water splitting reactions, or in the green production of a variety of synthetic hydrocarbons from biomass. All these applications require larger spot sizes that are compatible with their microstructure.

Applications in solution chemistry, such as understanding reaction kinetics or detection of intermediates, mostly require time resolution in the millisecond scale or faster that went beyond the capabilities of the available detection systems. Many interesting experiments in electrochemistry were confronted with a similar problem. These involve the search for better anode materials in electrochemical industrial processes for water electrolyses, cathodic protection, metal electrotwinning or for advanced photoanodes, and studies of kinetics and mechanisms of different reactions taking place at the electrodes (Reddy *et al.*, 2013 ▸). Another field of application of fast time-resolved XAS is in photochemistry studies. These have been of major interest in the last 30 years due to potential applications in solar energy conversion and storage, chemical sensing, photocatalysis and molecular devices. Metal coordination complexes and their photochemical and photophysical properties have been investigated in extensive detail, and, here too, timescales fall far beyond the millisecond. For all these applications, new detectors for EDXAS were required.

This paper describes the new EDXAS beamline ID24 within the TEXAS facility. §2 gives an overview of this beamline, from the source, to the optical scheme and detection systems. One sub-section is devoted to introducing the basic characteristics of the energy-dispersive spectrometer, such as the energy bandwidth of the polychromatic fan and energy resolution. §3 describes the experimental facilities available for users. In §4 we give a few examples of recent scientific results and conclusions are given in §5.

## 2. Beamline overview   

### 2.1. The ID24 beamline within the TEXAS facility   

**Figure 2:**
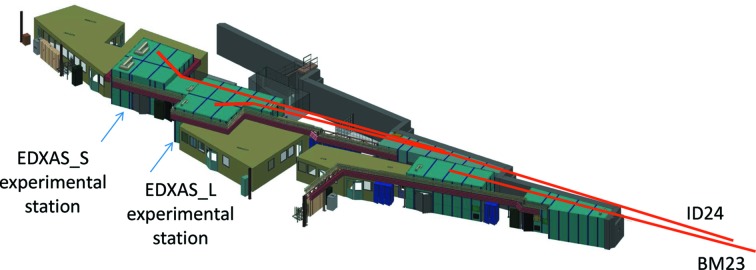
Layout of the TEXAS facility.

The TEXAS facility is illustrated in Fig. 2 ▸. It is composed of a 70 m-long EDXAS beamline on port ID24, coupled to a 40 m-long general purpose extended X-ray absorption fine structure (EXAFS) beamline on bending-magnet port BM23 (Mathon *et al.*, 2015*a* ▸).

The concept of combining a general purpose EXAFS beamline and a state-of-the-art high-brilliance EDXAS beamline in a single facility provides the user community with the best possible conditions for the research outlined in Fig. 1 ▸. BM23 is the only public ESRF beamline dedicated to ‘standard’ EXAFS. Its key mission is to provide high-quality EXAFS in a large energy range (5–75 keV), with high signal to noise (S/N) up to large *k*-range, with a high degree of automation, featuring online EXAFS data reduction and a flexible sample environment. The recent implementation of continuous scanning (quick-EXAFS) and of a micro-XAS station on BM23 provides a link between the time-resolved and extreme-conditions activities on BM23 and ID24. The possibility to achieve a focal spot of 4 µm × 4 µm on BM23, coupled to the recent availability of nano-polycrystalline diamond (NPD) anvils (Irifune *et al.*, 2003 ▸) is a real breakthrough for high-pressure XAS investigations. High-quality large *k*-range EXAFS can be recorded on BM23 in static mode or with mild time-resolution, be it at ambient conditions, at high pressure in a diamond anvil cell or *in situ* and *in operando* in a chemical reactor. These data are often of paramount importance to complement lower quality data (lower S/N, smaller *k*-range, *etc*.) recorded under more challenging conditions on ID24. For example, the local structure in the initial and final state of a system undergoing a fast reaction can be elucidated from BM23 data, whereas the signature of a short-lived intermediate state can be detected from ID24 data (Tromp *et al.*, 2013 ▸). The two beamlines BM23 and ID24 therefore complement each other, and together provide comprehensive experimental capabilities for time-resolved and extreme-conditions XAS.

### 2.2. Source   

The ID24 source is located on a 6 m-long high-β straight section of the storage ring. The magnetic sequence is formed by a combination of 27 mm (U27) and 32 mm (U32) period undulators as follows: U27 1.6 m + U27 1.4 m + Revolver U27/U32 1.4 m + U32 1.6 m. Table 1 ▸ lists the emittance values, the electron beam dimensions and divergence and size of the X-ray beam at 30 m from the source, all in r.m.s. values.

**Figure 3:**
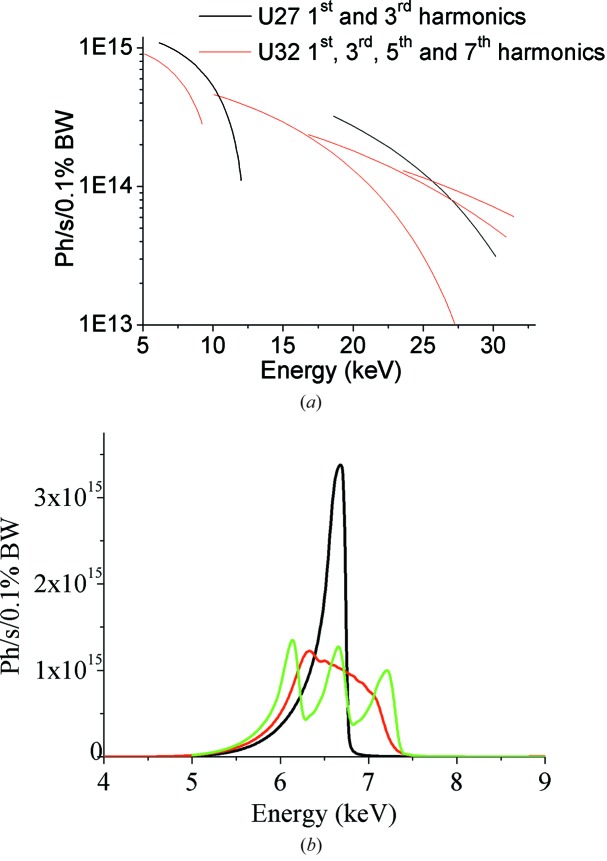
(*a*) Photons s^−1^ (0.1% bandwidth)^−1^ at 200 mA delivered by 1.6 m U32/U27 undulators over the beamline energy range 5–28 keV. (*b*) Effect of tapering. Using three tapered U27 undulators, an energy range of ∼1 keV can be achieved at 7 keV. Black: the three U27 undulators are at gap = 11.5 mm, no taper. Green: gaps = 11.0 mm, 11.7 mm and 12.5 mm, no taper. Red: gap = 11.75 mm using a linear taper of 1.5 mm.

The beamline roughly spans an energy range from 5 to 28 keV, covering absorption edges of elements between titanium and uranium. Fig. 3 ▸ illustrates the photon flux emitted by each type of undulator for a machine current of 200 mA over the full energy range.

The required energy bandwidth for EXAFS is achieved by tapering the three undulators. Fig. 3(*b*) ▸ compares the flux from the first harmonic of the U27 undulators in three different configurations: same gap, no taper (black); different gap, no taper (green); linear taper of 1.5 mm applied on the three undulators (red). The latter provides an energy bandwidth of ∼1 keV at 7 keV.

### 2.3. Optical scheme   

**Figure 4:**
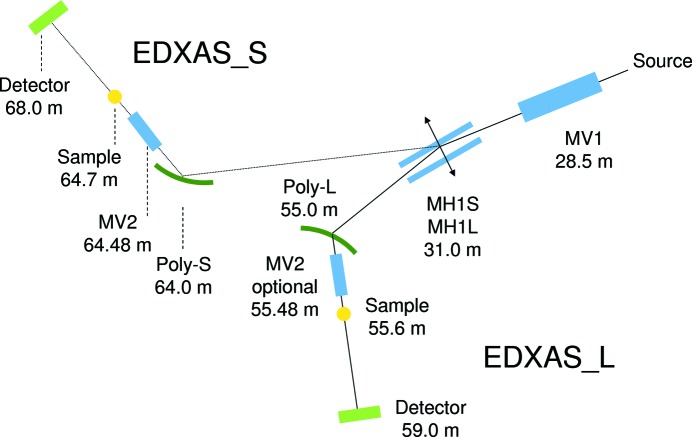
Beamline ID24 at the ESRF: the new design features two branches, EDXAS_S and EDXAS_L. Mirrors are in blue, polychromators in green, samples in yellow and detectors in light green. Acronyms MH1S, MH1L, MV1, MV2 are explained in the text.

In order to comply to the needs of the different user communities, without making compromises on the quality of the beam (*i.e.* spot size or energy range), the upgraded version of ID24 features two branches (Mathon *et al.*, 2010 ▸), EDXAS_S and EDXAS_L, as illustrated in Fig. 4 ▸, where the labels S and L stand for small and large spot applications, respectively. This distinction relies largely on the use of different polychromators, which determine the efficiency of the horizontal focusing. On branch EDXAS_S, an elliptically bent Si(111) or Si(311) crystal in Bragg geometry (Poly_S) delivers horizontal focal spots down to ∼3 µm FWHM. This geometry is ideally suited for the energy range 5–13 keV. At higher energies, penetration depth effects deteriorate both energy resolution and focal spot shape (Hagelstein *et al.*, 1995 ▸; Sanchez del Rio *et al.*, 2015 ▸). On branch EDXAS_L, a Si(111) polychromator crystal in Laue geometry (Poly_L) delivers larger spots, varying between 30 and 200 µm FWHM. This geometry covers applications at higher energies, roughly from 10 to 28 keV, that are not compatible with the Bragg geometry. The two branches are complementary, but the EDXAS_L polychromator chamber is designed to host both Laue and Bragg configurations and the ‘L’ branch hosts many ‘small spot’ and/or low-energy applications. The double branch scheme both enables best X-ray optical performance in terms of spot size and energy resolution for different applications, and also increases beam time efficiency as it allows complex experiments to be assembled, interfaced and tested on one branch while experiments are running on the other.

Besides the polychromator, the optical scheme consists of a pair of mirrors (MV1 and MH1) in a Kirkpatrick–Baez configuration, and an optional vertically refocusing mirror (MV2). The optical configurations in the vertical and horizontal planes are discussed in §2.4 and §2.6, respectively.

### 2.4. Vertical focusing   

**Figure 5:**
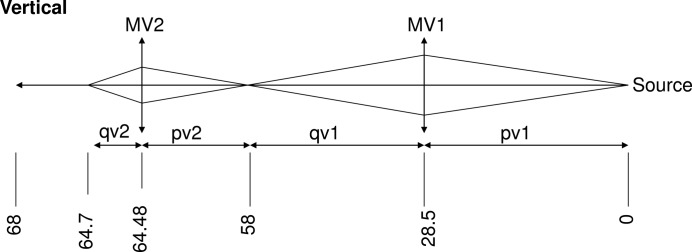
Optical scheme of the EDXAS_S branch in the vertical plane.

The radius of curvature of mirror MV1 can be adjusted either to focus directly on the sample (mostly for EDXAS_L), or to create a secondary source that is then refocused by MV2 for micro-XAS applications (mostly for EDXAS_S). Fig. 5 ▸ illustrates the vertical plane optical scheme adopted for branch EDXAS_S.

**Figure 6:**
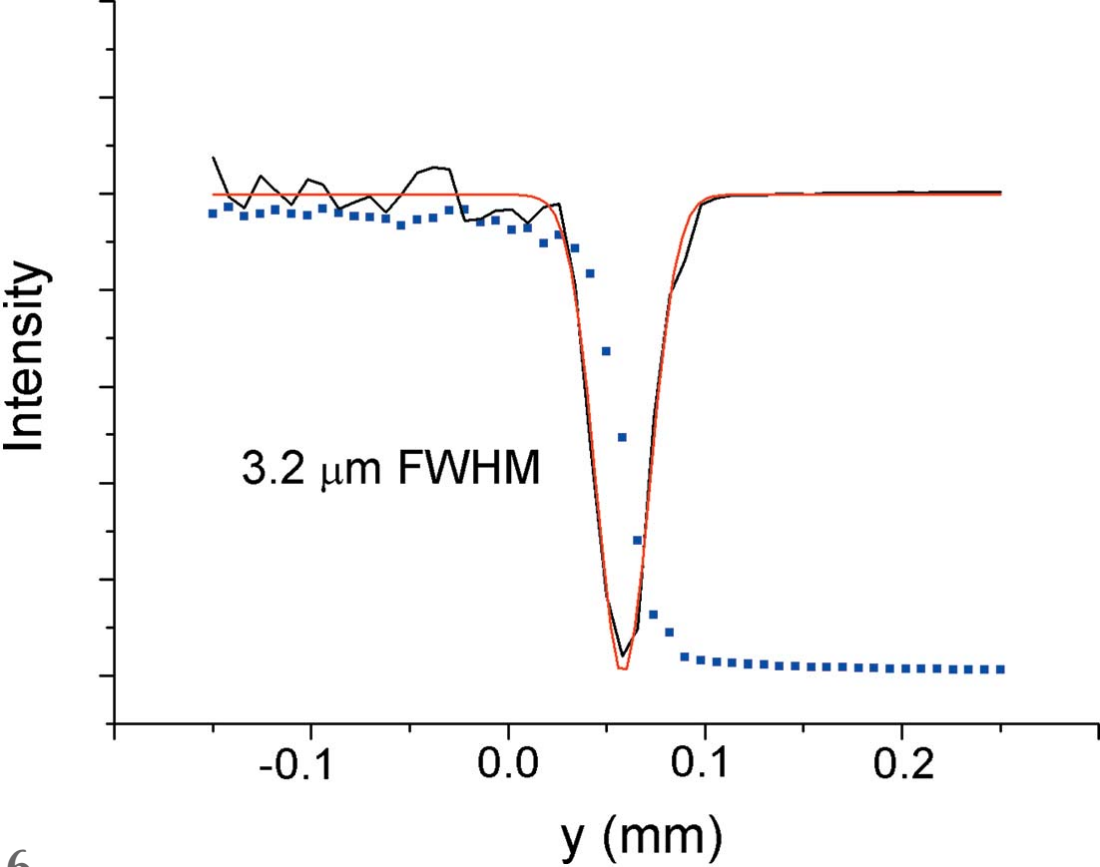
Knife-edge scan (blue dots) on the sample position along the vertical direction. A Gaussian fit (red line) to the derivative (black line) yields a spot size of 3.2 µm FWHM.

Fig. 6 ▸ shows the knife edge and its derivative at the 64.7 m focus. The FWHM value achieved is 3.2 µm, fully consistent with ray-tracing calculations which include source size and MV1 and MV2 slope error contribution.

### 2.5. Bandwith, resolution and flux   

**Figure 7:**
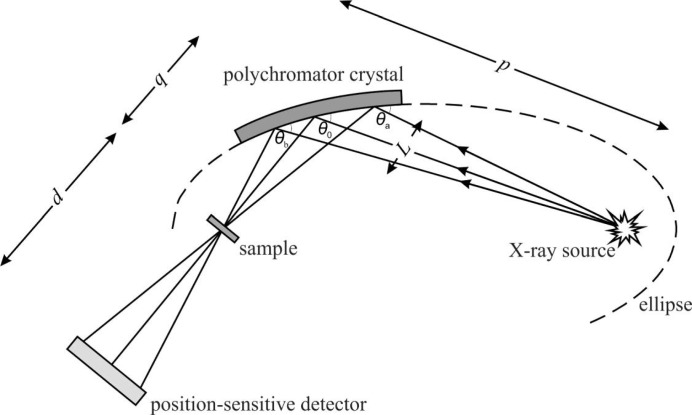
Principle of operation of the energy-dispersive XAS spectrometer. On beamline ID24, the X-ray source in this figure is a demagnified image of the undulator source (*i.e.* secondary source).

The energy-dispersive spectrometer employs a curved crystal to focus an angle-dispersed polychromatic fan of X-rays onto the sample position (Matsushita & Phizackerley, 1981 ▸). As shown in Fig. 7 ▸, the transmitted beam is detected by a position-sensitive detector positioned at the end of the 2θ spectrometer arm where energy is correlated to position.

The main advantages of EDXAS spectrometers with respect to energy scanning spectrometers are: (i) the speed of acquisition, since all energy points are acquired simultaneously, and (ii) the stability of the energy scale and focal spot position, since there are no moving components during acquisition.

There are two main limitations that are intrinsic to EDXAS: (i) a need to preserve the energy–direction correlation created by the polychromator from the crystal to the detector which restricts the morphology and microstructure of suitable samples, and (ii) the impossibility of using de-excitation processes (fluorescence, total electron yield, *etc*.), since all energies impinge on the sample simultaneously. These limitations have influenced the development of EDXAS, and, although this technique is now generally available at major synchrotron sources, the EDXAS user community remains a minor portion of the global XAS user community. Reviews of techniques and applications of EDXAS may be found by Pascarelli *et al.* (2006 ▸; microXAS), Aquilanti *et al.* (2009*b* ▸; high pressure), Pascarelli & Mathon (2010 ▸; high brilliance applications), Torchio *et al.* (2014*b* ▸; magnetism at extreme conditions) and Mathon *et al.* (2015*b* ▸; time-resolved applications).

#### 2.5.1. Energy bandwidth   

**Figure 8:**
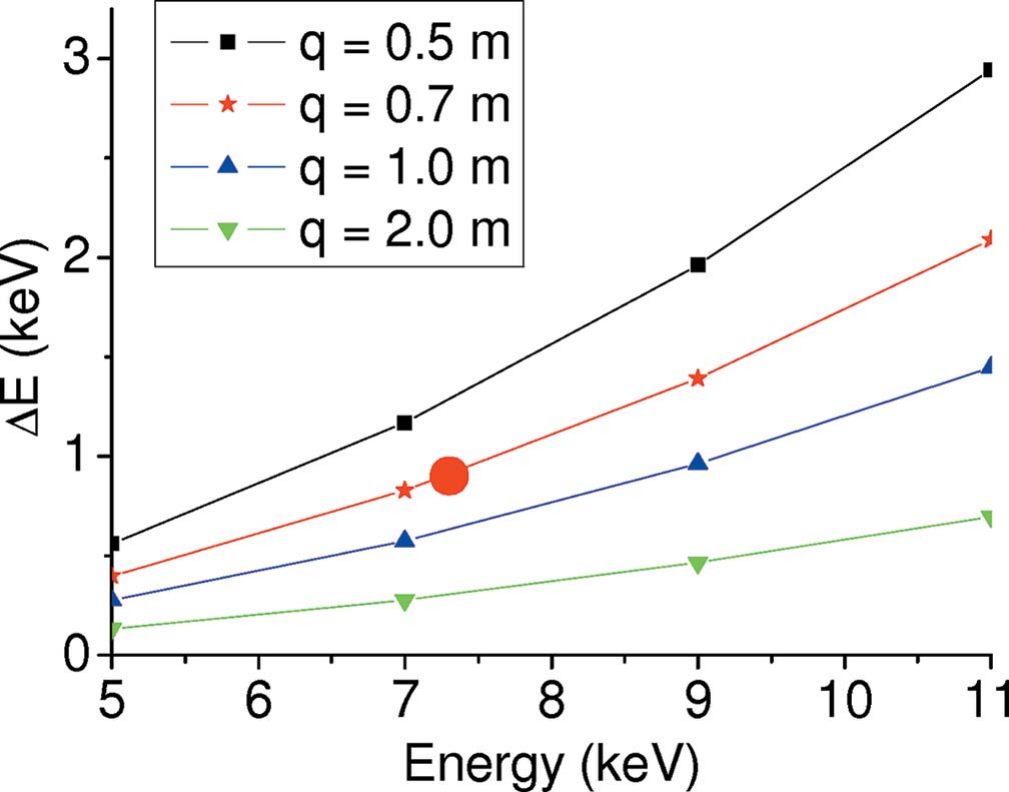
Full spectral range Δ*E* diffracted by a Si(111) polychromator as a function of central energy *E*_0_, from equations (1) and (2), calculated for branch EDXAS_S (*L* = 50 mm and *p* = 29.7 m). The red dot marks the measured Δ*E* value at the Fe *K*-edge, with *q* = 0.7 m.

The crystal is curved to an elliptical shape. The local radius is *R* = 2*pq*/(*p* + *q*)sinθ_0_. Here, *p*, *q* and θ_0_ are the source–crystal and crystal–sample distances and central Bragg angle, respectively. In Fig. 7 ▸, *L* is the horizontal dimension of the beam intercepted by the polychromator, and *L*/sinθ_0_ is the footprint of the beam on the crystal surface. The full spectral range diffracted by the crystal, Δ*E*, is proportional to the variation of Bragg angle θ along the beam footprint on the crystal, Δθ:
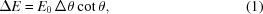
where *E*_0_ is the central energy. Δθ can be calculated from *p*, *q* and *L* as follows:
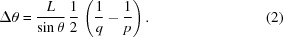



In general, EDXAS spectrometers are installed on synchrotron sources having a large horizontal divergence, leading to a large beam footprint on the surface of the crystal that provides a sufficiently large energy bandwidth to cover a full EXAFS spectrum in a single shot. Equation (2) is valid as long as *L*/sinθ is smaller than the useful length of the polychromator crystal. Equation (1) highlights an additional important limitation of EDXAS: at low energies the full spectral range diffracted by the polychromator Δ*E* is strongly reduced due to the cotθ factor. This is illustrated in Fig. 8 ▸, which shows typical Δ*E* *versus* *E*_0_ values for different *q* values on ID24.

For a fixed choice of *E*_0_, Δ*E* strongly depends on the polychromator–sample distance *q*. An important upgrade effort was put into redesigning the area around the polychromators in order to reduce *q* as much as possible, with the aim to increase the energy range of the diffracted polychromatic beam at low energies. The minimum focal distance is ∼0.7 m, leading to Δ*E* ≃ 900 eV (*k*_max_ ≃ 12 A^−1^) at the Fe *K*-edge allowing an, albeit limited, EXAFS analysis to be performed. The red circle in Fig. 8 ▸ compares the Δ*E* value measured at the Fe *K*-edge (central energy 7. 3 keV) and *q* ≃ 0.7 with the expected trend.

#### 2.5.2. Energy resolution   

**Figure 9:**
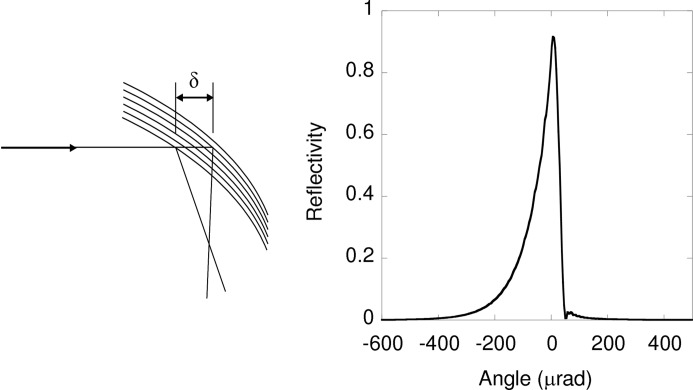
Left: schematic effect of X-ray penetration in a curved crystal. Right: simulation of the diffraction profile deformation of Si(111) crystal at 15 keV due to a 3 m radius of curvature.

**Figure 10:**
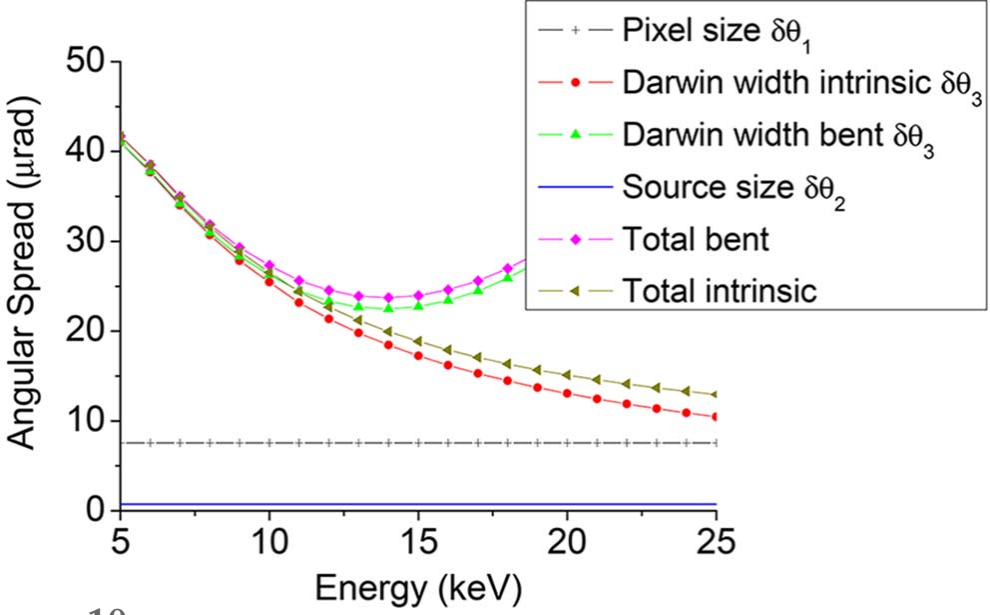
Angular contribution to the energy resolution *versus* nominal energy *E*_0_, *q* = 0.7 m, detector at *d* = 3.3 m for a Si(111) Bragg crystal.

The energy resolution of an energy-dispersive spectrometer depends on the central energy, *E*_0_, the crystal diffracting planes (*h*, *k*, *l*), the focusing distance *q* and detector position *d*. The energy resolution repeats equation (1), but with Δθ having new significance: Δθ is now the ‘effective’ angular spread δθ. For the Bragg geometry, the effective angular spread includes three contributions δθ_1_, δθ_2_ and δθ_3_ that derive respectively from: (i) the spatial resolution of the detector, (ii) the size of the X-ray source and (iii) the Darwin width of the curved polychromator crystal:
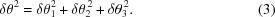



On ID24, δθ_2_ is negligible (see Fig. 10 below). The term δθ_1_ is proportional to δ*r*/*d*, where δ*r* is the point spread function of the detector and is a function of the pixel horizontal size. The latter varies between 15 and 50 µm, depending on the detector used. The incident beam intercepts Bragg planes with a continuously different angle when it penetrates the crystal leading to an asymmetrical reflectivity profile of the highly curved Bragg-type crystal. The effect can be simulated (Sanchez del Rio *et al.*, 2015 ▸) and is illustrated in Fig. 9 ▸. This degrades energy resolution and creates an asymmetrical shape of the focal spot (see §2.6 below). This contribution increases with energy and with crystal curvature. It is small at 7 keV and becomes dominant at 18 keV for a Si(111) crystal. This sets the fundamental limit of the Bragg geometry for focal spot size and energy resolution at high energies (>13 keV).

**Figure 11:**
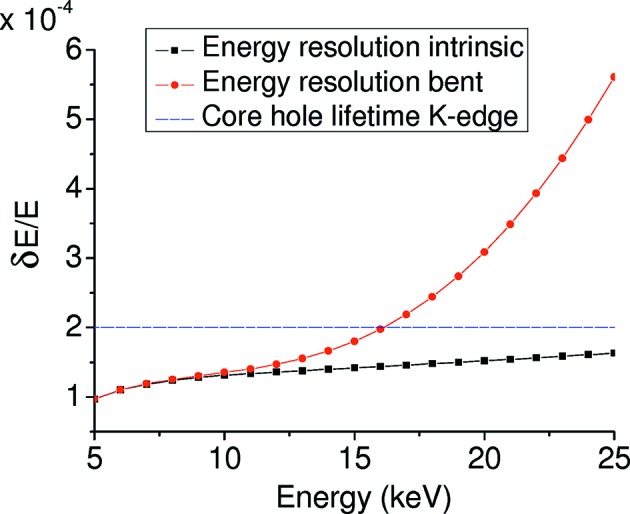
Energy resolution *versus* nominal energy *E*_0_, *q* = 0.7 m, detector at *d* = 3.3 m for a Si(111) Bragg crystal.

Figs. 10 ▸ and 11 ▸ report, respectively, the angular contributions δθ_i_ and the energy resolution δ*E*/*E* for a Bragg Si(111) crystal focusing at *q* = 0.7 m with a detector at *d* = 3.3 m. These figures show that the main contributions derive from δθ_3_ and δθ_1_ at low and high energies, respectively. In particular, Fig. 10 ▸ shows the increase of the spread of the Darwin width with energy, while Fig. 11 ▸ illustrates that for energies larger than 15 keV the energy resolution of the bent Bragg crystal exceeds the *K*-edge core-hole lifetime contribution. This effect, together with the geometric reduction in horizontal acceptance of the crystal at low Bragg angles, limits the use of the Bragg geometry to the range 5–13 keV approximately.

In the bent symmetric Laue case, the deformation induced by bending is orthogonal to the reciprocal lattice. The case can be treated as an unbent crystal and, contrary to the Bragg case, there is no broadening of the Darwin width whatever the bending radius is. Consequences are that there is no broadening of the focal spot size nor a loss in energy resolution at high energy. δθ in equation (4) includes four contributions δθ_1_, δθ_2_, δθ_3_ and δθ_4_ that derive, respectively, from: (i) the spatial resolution of the detector, (ii) the size of the X-ray source, (iii) the intrinsic Darwin width of the curved Laue polychromator crystal and (iv) the spread of the monochromatic beam on the detector due to the Borrmann fan,
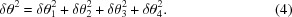
The main contributions derive from δθ_3_ and δθ_4_ at low energies and δθ_1_ at high energies.

**Figure 12:**
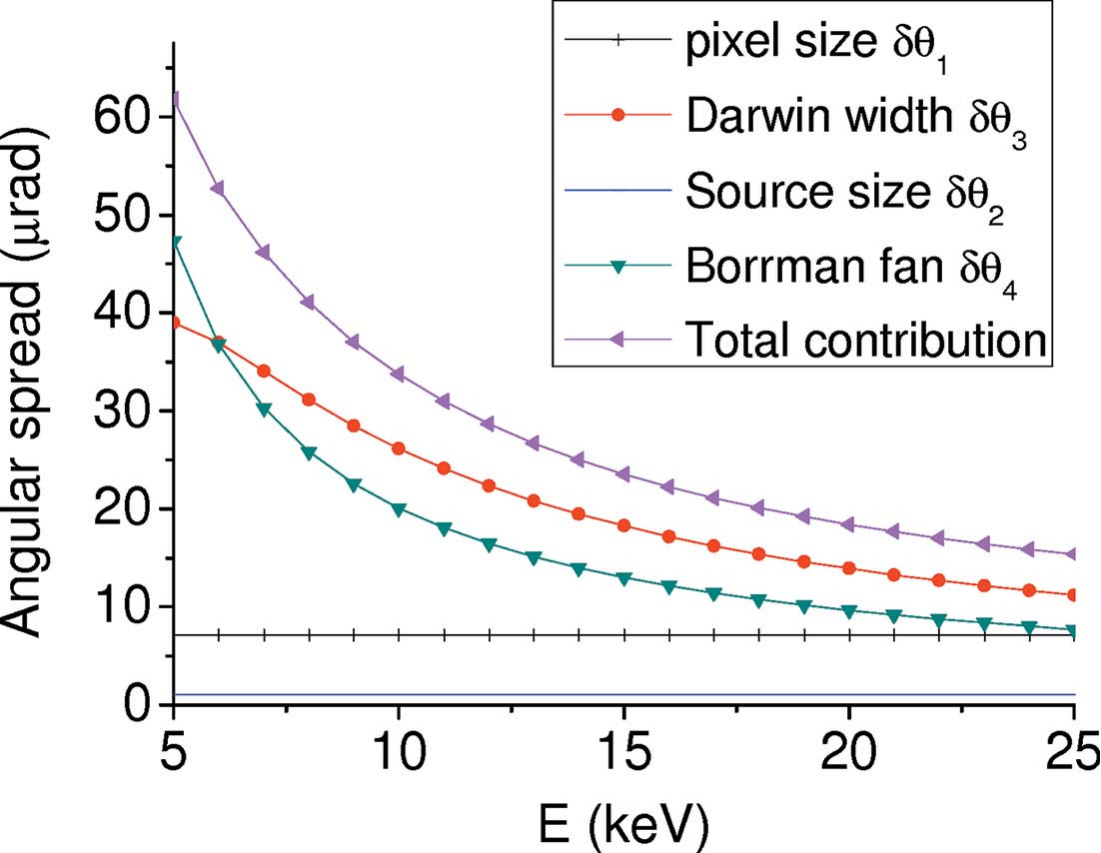
Angular contributions to the energy resolution for a 200 µm-thick Si(111) Laue crystal focusing at *q* = 0.6 m with a detector at *d* = 3.4 m.

**Figure 13:**
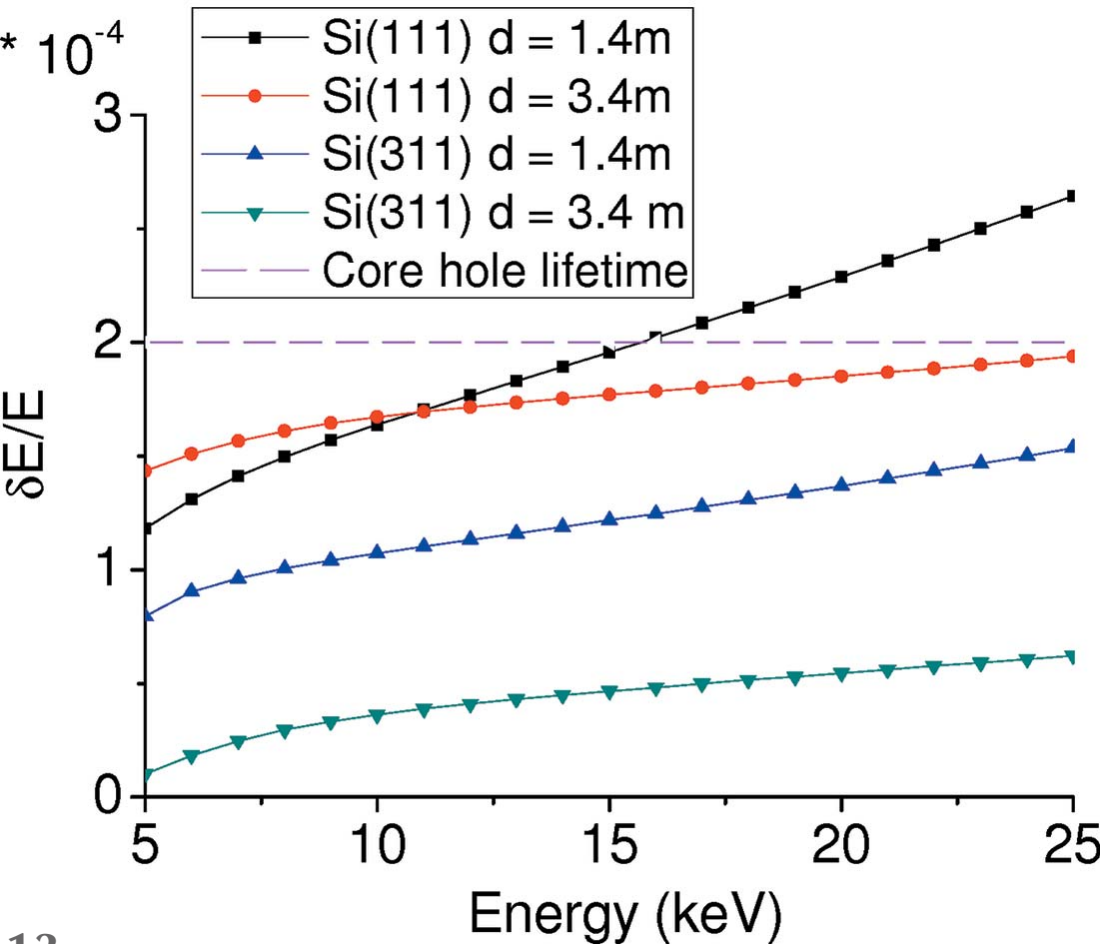
Energy resolution *versus* nominal energy, *q* = 0.6 m, detector at *d* = 1.4 m and *d* = 3.4 m, for Si(111) (squares and circles) and Si(311) (up and down triangles) Laue crystals.

Fig. 12 ▸ shows the angular contributions δθ_*i*_ to the energy resolution for a 200 µm-thick Laue Si(111) crystal focusing at *q* = 0.6 m with a detector at *d* = 3.4 m. Fig. 13 ▸ presents the energy resolution δ*E*/*E* for a focusing distance of *q* = 0.6 m with a detector at *d* = 1.4 m and *d* = 3.4 m, for Si(111) and Si(311) crystals.

#### 2.5.3. Flux on the sample   

**Figure 14:**
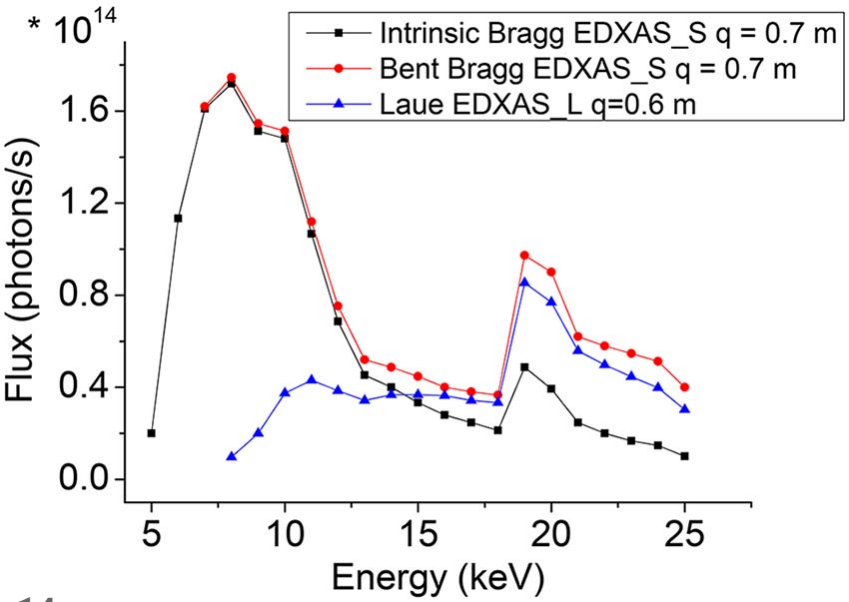
Flux on sample for a Si(111) crystal. Squares: EDXAS_S, Bragg geometry (intrinsic Darwin width), *q* = 0.7 m. Circles: EDXAS_S, Bragg geometry (bent Darwin width), *q* = 0.7 m. Triangles: EDXAS_L, Laue geometry, *q* = 0.6 m. Between 13 and 18 keV, only the two U32 undulators contribute to the flux.

Fig. 14 ▸ presents the flux expected on the sample for a Si(111) crystal in Bragg geometry (intrinsic and bent) focusing at a distance of *q* = 0.7 m. The calculations include four undulators (only two undulators U32 are available between 13 and 18 keV) at 200 mA, the transmission of the diamond and Be windows along the beam path, the reflectivity of the two KB mirrors (MV1 and MH1), the reflectivity of the Si(111) polychromator, the limitation of the geometrical acceptance at high energy and the reflectivity of MV2. The flux profile peaks at energies covering the *K*-edges of the 3*d* metals (6–10 keV) where a very large portion of the experiments are carried out. The figure also reports the flux expected on the sample for a Si(111) crystal in Laue geometry focusing at a distance of *q* = 0.6 m. The calculation includes four undulators (only two U32 undulators between 13 and 18 keV) at 200 mA, the transmission of the diamond and Be windows along the beam path, the reflectivity of MV1 and MH1, and the reflectivity of the 200 µm-thick bent Si polychromator in Laue geometry. The strong reduction in flux at low energy, coupled to heat load considerations, limits the practical use of the Laue geometry to energies above ∼10 keV.

### 2.6. Horizontal focusing   

**Figure 15:**
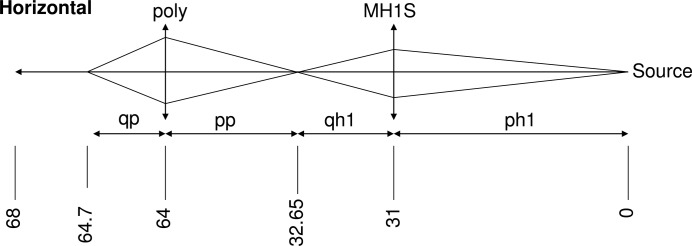
Optical scheme of the EDXAS_S branch in the horizontal plane.

**Figure 16:**
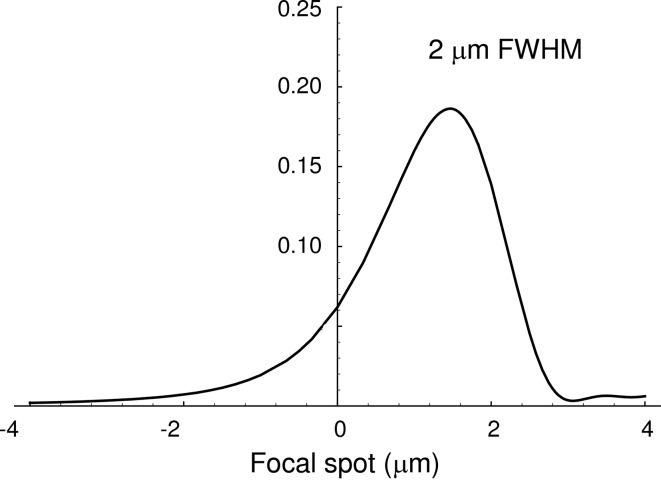
Contribution to the focal spot size for a Si(111) crystal at 7 keV with *p* = 30 m and *q* = 1 m.

Fig. 15 ▸ illustrates the horizontal plane optical scheme adopted for branch EDXAS_S. The role of MH1 is to transform the highly collimated undulator beam into a diverging beam in the horizontal plane (Hagelstein *et al.*, 1997 ▸), to provide a large footprint on the crystal and therefore a large energy range suitable for EXAFS in the polychromatic fan [Δ*E*, see equation (1) in §2.5]. This mirror creates a secondary source at 32.65 m which is then refocused by the polychromator. Focusing using bent Bragg planes introduces an angular spread. There are two contributions: (i) the Bragg angles vary as a function of penetration depth, due to the bent shape of the crystal (see Fig. 9 ▸), and (ii) in the Bragg diffraction condition, a crystal (bent or flat) has a natural angular acceptance (Darwin width) ω_D_ when highlighted by a monochromatic divergent beam. This leads to a monochromatic diffracted beam with a natural divergence Δθ = 2ω_D_. Due to the focusing diffraction limit, such a beam leads to an intrinsic transverse focal spot size Δ*x* of
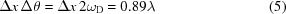
if the diffraction profile is approximated by a rectangle. At 7 keV for a Si(111) crystal the intrinsic dynamical contribution to the focal spot size is Δ*x* ≃ 2 µm. This result has been confirmed by full dynamical theory simulations of the Bragg bender case (Mocella, 2009 ▸) as shown in Fig. 16 ▸. For a polychromatic incident beam, the final spot size is the result of the sum of all monochromatic contributions. Therefore focusing a polychromatic incident divergent beam with a bent crystal leads to an intrinsic spot size limit of the order of 2 µm FWHM in the Bragg geometry.

In the horizontal plane, another limiting factor to the spot size is the slope error of the Bragg polychromator crystal. The difficulties here are related to the manufacture of the 300 mm × 20 mm 2 mm-thick Si crystals, the thickness of which needs to remain constant to within a few micrometers along the full length. New optimized profiles for the Bragg crystals have been specifically designed by finite-element analysis (Zhang, 2009 ▸) with expected slope error of 0.7 µrad peak-to-peak (PP) over the central 200 mm. The protocol for the manufacture of such crystals is constantly being improved but the focal spot sizes still depend on the portion of the polychromatic fan (or on the portion of the illuminated footprint on the crystal) that is selected.

**Figure 17:**
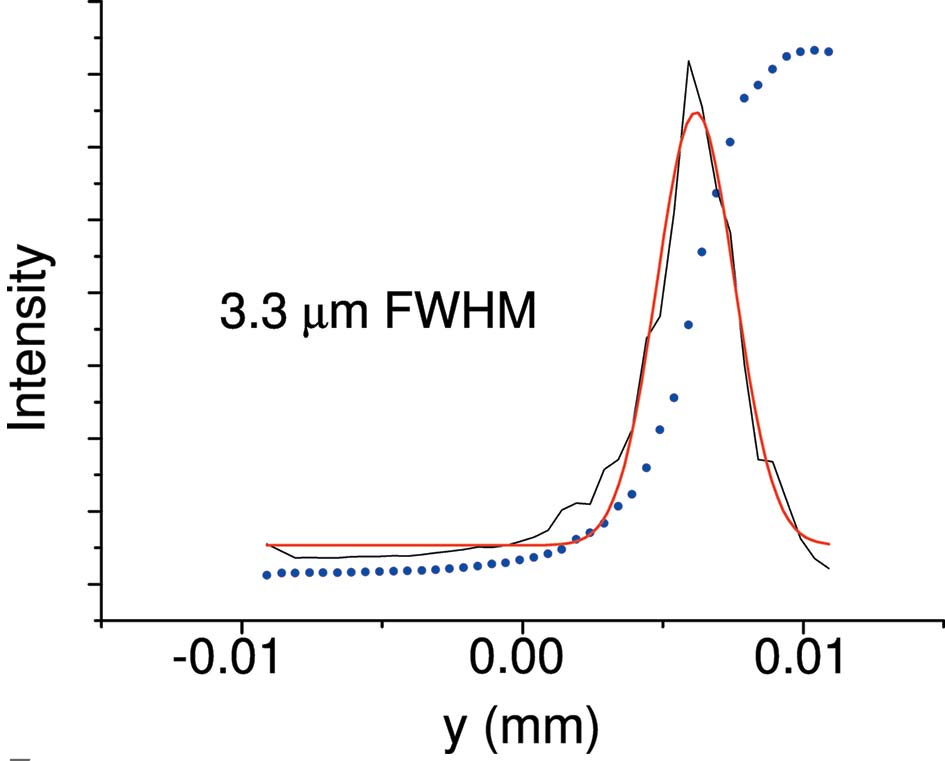
Knife-edge scan (blue dots) on the sample position along the horizontal direction. A Gaussian fit (red line) to the derivative (black line) yields a spot size of 3.3 µm FWHM.

In routine operation on EDXAS_S, at the Fe *K*-edge and at *q* ≃ 0.75 m, the focal spot achieved is ∼4 µm × 4 µm FWHM with a selected energy range of ∼500 eV, although the full spectral range diffracted by the polychromator in this geometry is Δ*E* ≃ 800 eV [from equation (1)]. The accuracy of the crystal bending and therefore the bender and crystal preparation technology is a key point of the EDXAS spectrometer. Figs. 17 ▸ and 18 ▸ illustrate, respectively, a horizontal knife-edge scan on the focal plane at *q* ≃ 0.75 m and an example of EXAFS recorded on an Fe foil sample within a diamond anvil cell under these conditions.

### 2.7. Stability   

Thermal stability of optical elements (mirrors and polychromator) affects slow (<1 Hz) beam movements. The total power deposited on MV1 under the most severe working conditions (*I* = 200 mA, low energies) is of the order of 800 W. A number of thermal strategies have been adopted ranging from installing Compton scatter shields and minimizing components inside the mirror vacuum chamber and keeping a constant heat load on the mirror by adjusting the high-power primary slits horizontal gap proportionally to the current in the machine. The radius of curvature of MV1 is also adjusted as a function of thermal load. To reduce thermal bump effects, secondary high-power vertical slits positioned downstream of MV1 allow to overflood this mirror and select only the portion of the beam reflected by the central part of its surface. Typical values of high-power primary and secondary slits are 3.0 mm × 1.5 mm and 2.5 mm × 1.0 mm (H × V), respectively.

Thermal load on the polychromator is at most 350 W total power, with a maximum power density of 1.5 W mm^−2^. Generally this is well controlled in the case of the Bragg polychromator. For the Laue case, at low energies a large portion of the incident flux is absorbed by the crystal leading to strong thermal instabilities. The latter are controlled by a careful choice of undulator gap and attenuators, to reduce unwanted low-energy photons while maximizing the flux around the selected absorption edge. It is mainly for this reason that the use of the Laue geometry is generally restricted to *E* > 10 keV.

**Figure 19:**
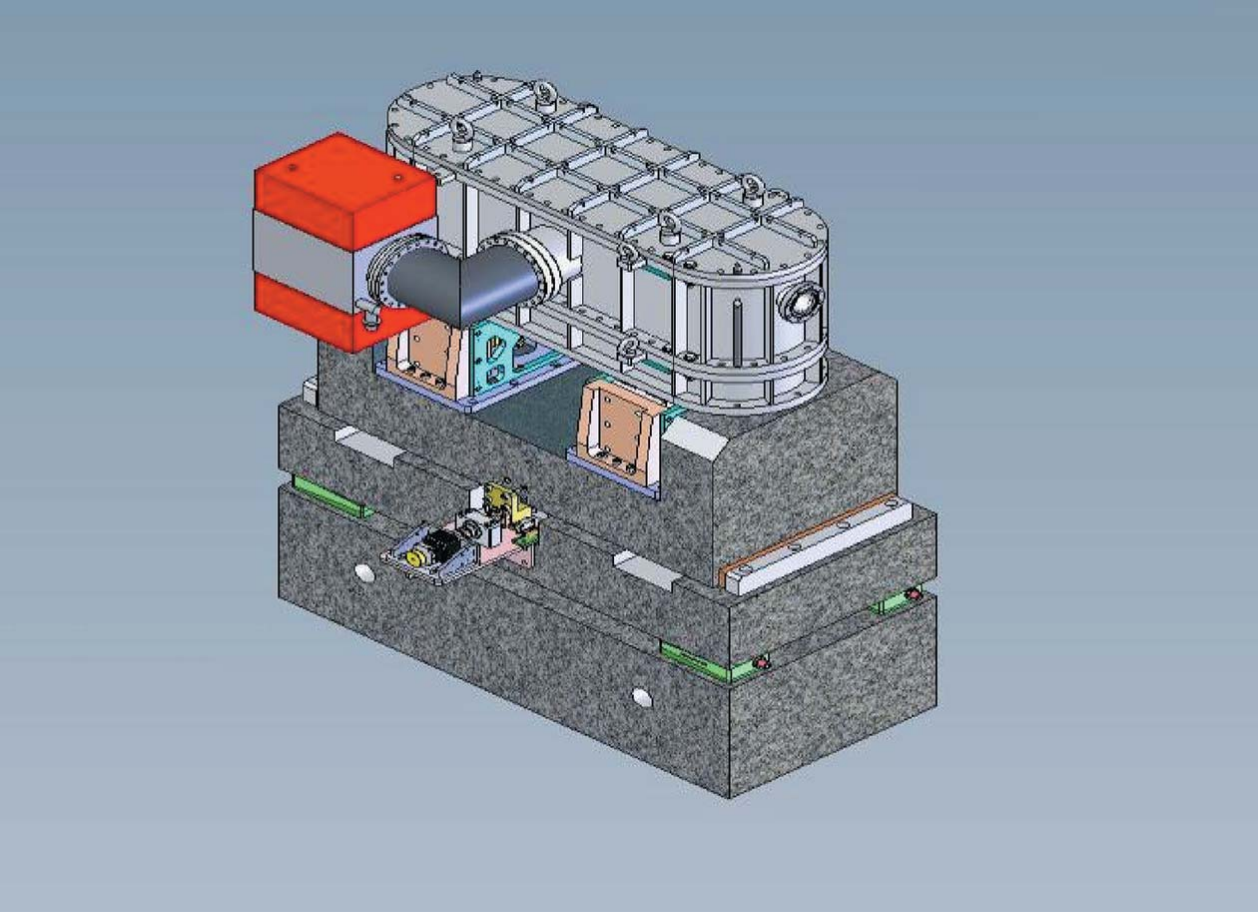
The design of the new support for MV1 features three successive granite blocks and highly accurate and ultra-stable mirror positioning systems.

**Figure 20:**
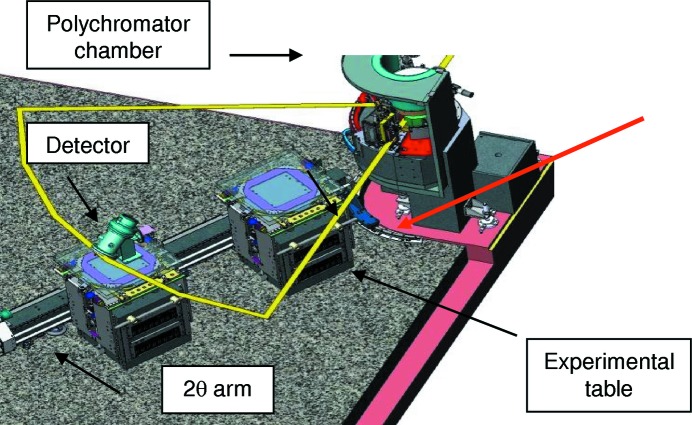
Schematic drawing of the experimental hutch on branch EDXAS_L. The granite floor covers the full surface of the hutch. The experimental table, the detector support and the 2θ arm are moved on airpads mounted on granite chassis. The 2θ arm is used only as a guide. The red arrow indicates the X-ray propagation direction.

Mechanical stability, again mainly of MV1, affects higher frequency (>Hz) beam movements. The design of the new support for MV1 features three successive granite blocks (Fig. 19 ▸) and highly accurate and ultra-stable mirror positioning systems (Baker *et al.*, 2013 ▸). To reduce vibration levels, granite supports are used for all optical elements, sample environments and detection systems and granite slabs form the floors in the two experimental hutches. Every experimental setup is supported by granite tables which are placed into and out of the beam by means of air pads (see Fig. 20 ▸).

**Figure 21:**
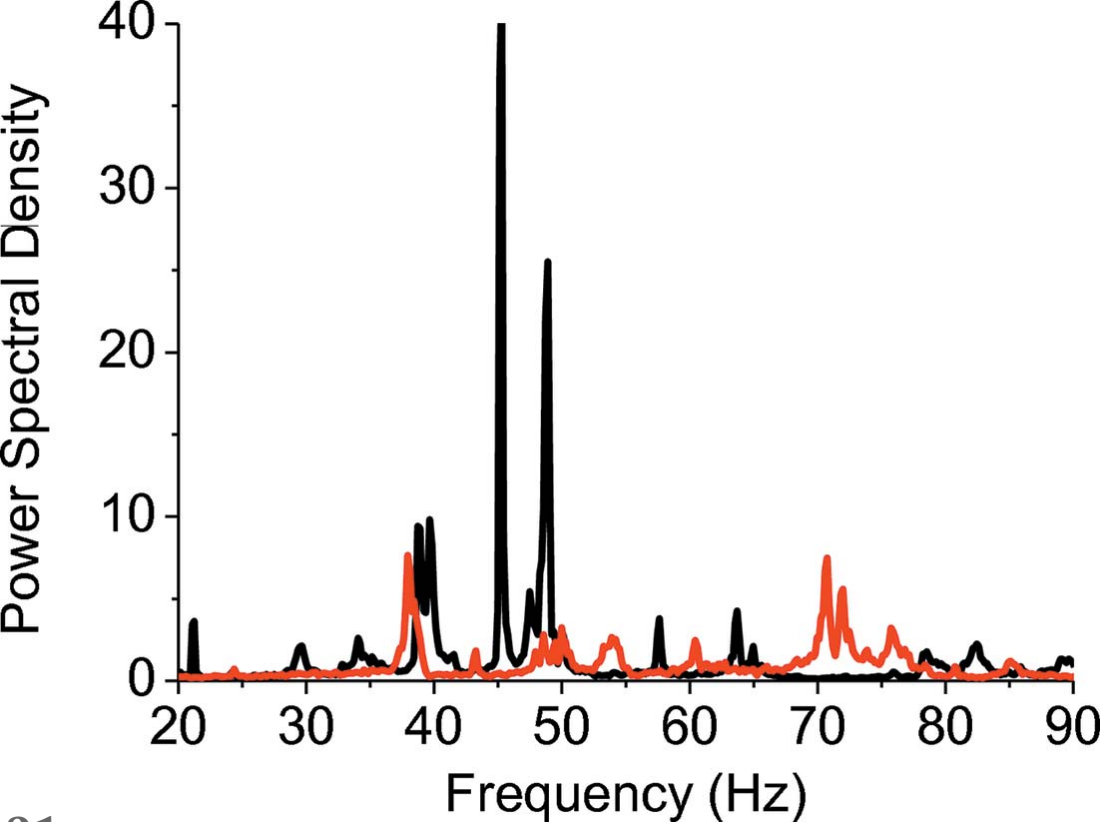
Power spectral density of beam instabilities in the vertical plane, measured on the focal spot, in 2008 (black) and 2011 (red). The reduction in the amplitude of vibrations between 30 and 50 Hz is visible.

A substantial reduction of the PP amplitude of beam instabilities in both the horizontal and vertical directions has been observed, as illustrated in Figs. 21 ▸ and 22 ▸.

### 2.8. Detection   

**Figure 23:**
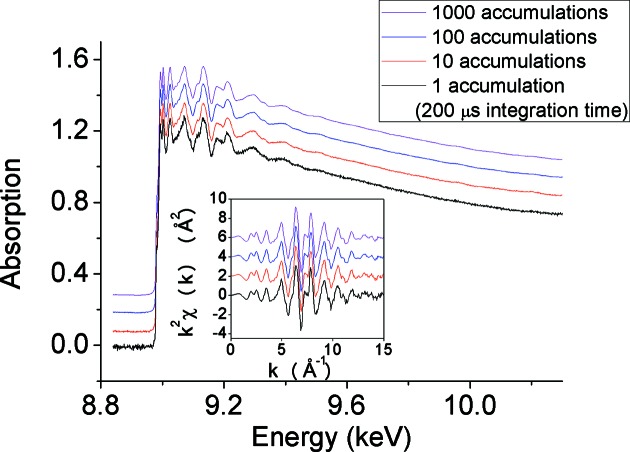
Sequence of Cu *K*-edge EXAFS acquired with the new Hamamatsu chip. From bottom to top: average of 1, 10, 100, 1000 accumulations of 200 µs each. The accumulated curves are offset from the single accumulation for clarity. The inset shows the *k* ^2^χ(*k*) signals. Taken from Kantor *et al.* (2014 ▸).

One of the most important issues addressed by the upgrade was related to the urgent need to move towards faster readout time while maintaining a high dynamic range, linearity and spatial (or, equivalently, energy) resolution. Two major upgrades were achieved on the home-made two-dimensional FReLoN camera (Labiche *et al.*, 2007 ▸): (i) an increase in the field of view of the optical interface, from 50 mm to 100 mm, which became necessary in order to maintain the required energy resolution and total energy range, following the reduction in focal distance *q*, and (ii) an exchange of the two-dimensional CCD camera with a ∼10 kHz one-dimensional commercially available Hamamatsu chip (Kantor *et al.*, 2014 ▸). Fig. 23 ▸ illustrates an example of the performance of this new detection system. In addition, a major effort was made to develop, in collaboration with the Science & Technology Facility Council (STFC), a new ‘X-ray harder’ position-sensitive direct detection system capable of pushing acquisition frequency towards the MHz range. The new Ge strip detector, based on a previous design (Headspith *et al.*, 2007 ▸) but equipped with new acquisition electronics, features a readout time of 2.8 µs and minimum integration time of ∼80 ns. First results are reported in §4.

## 3. Experimental facilities   

TEXAS includes the infrastructure for a wide range of experiments. Different sample environments have been implemented in the two experimental hutches EDXAS_S and EDXAS_L, targeting different user communities.

### 3.1. The EDXAS_S branch   

The EDXAS_S branch hosts facilities for experiments at extreme conditions of pressure and temperature and micro-XAS hyperspectral mapping. This branch attracts mainly the geo- and environmental science user communities.

#### 3.1.1. The *in situ* laser heating facility   

**Figure 24:**
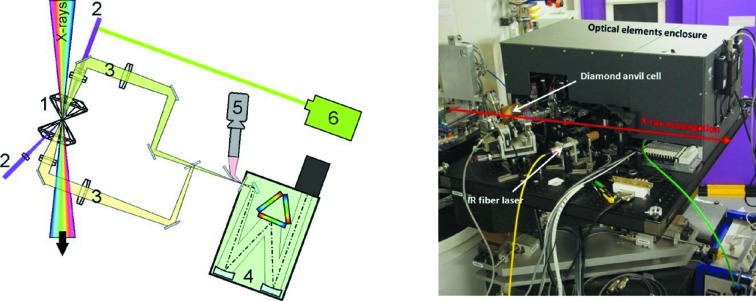
Schematic drawing (left) and photograph (right) of the *in situ* laser-heating facility for high-pressure studies with the DAC. 1: DAC; 2: high-power IR laser beams; 3: emitted radiation from hot sample; 4: spectrometer; 5: CCD video camera and microscope; 6: green laser for Raman spectroscopy.

One of the scientific drivers of the upgrade of ID24 was the *in situ* investigation of the electronic and local structure of matter at pressure and temperature relevant to the Earth’s interior. It is now well established (see, for example, Salamat *et al.*, 2014 ▸) that these conditions are best achieved using double-sided laser heating of samples in a diamond anvil cell (DAC). This had never been achieved before by XAS, and requires the combination of a small, stable focal spot and fast acquisition. An *in situ* laser-heating facility for high-pressure applications with the DAC (Kantor *et al.*, 2012 ▸; Marini *et al.*, 2013 ▸) has therefore been specifically designed and is illustrated in Fig. 24 ▸. The double-sided laser-heating system is designed in such a way that the optical components do not interfere with the direct X-ray beam, allowing true simultaneous measurements of temperature from both sides of the sample as well as the X-ray absorption spectra.

The DAC is mounted into a water-cooled copper holder and is supported by a mini-hexapod stage. The sample within the DAC is heated by means of two 1070 nm 120 W IR fiber lasers, focused on both sides of the sample. The lasers operate either in pulsed (pulse width ≃ 0.1–100 ms) or in continuous mode. Fast data acquisition is required because laser-heated samples are unstable and can react with their environment. The sample temperature is measured by collecting and analyzing the emitted radiation from both sides of the sample using a Princeton Instruments SP300i spectrometer, and fitting it to Planck’s law within a grey body approximation. The sample image is collected by custom objectives and then projected onto the entrance of the spectrometer. A microscope coupled to a high-performance CCD video camera is used to visualize the sample and to perform sample alignment. The pressure is measured *in situ* from the shift of the fluorescence signal from a ruby, or by the shift of the Raman signal from the surface of the diamond culet, excited by a green Raman laser.

#### 3.1.2. The micro-XAS station for two-dimensional hyperspectral mapping   

**Figure 25:**
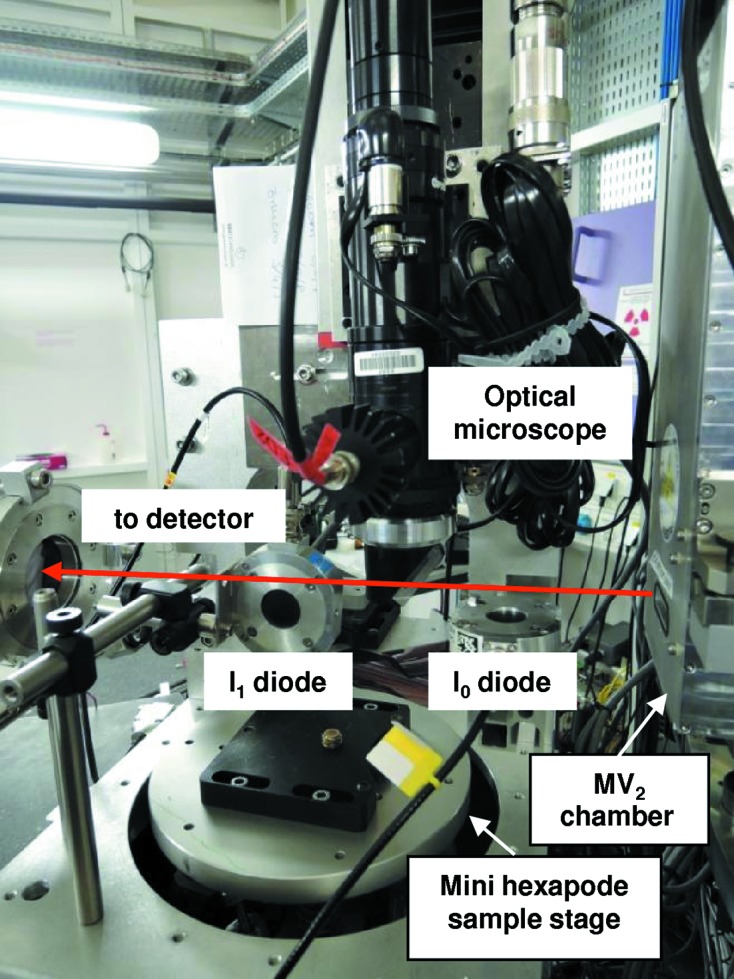
Photograph of the micro-XAS facility on EDXAS_S.

A micro-XAS station optimized for two-dimensional hyperspectral mapping of heterogeneous samples (Muñoz *et al.*, 2006 ▸) in transmission mode or in fluorescence mode using sequential acquisition (Pascarelli *et al.*, 1999 ▸) is available in EDXAS_S. This facility (shown in Fig. 25 ▸) features an optical microscope to visualize the portion of the sample to investigate and to align the sample in the beam. A small ion chamber or a Si PIN diode measure the incoming intensity *I*_0_. A Si PIN diode or a Si drift vortex detector measures the total fluorescence signal. The sample is mounted on a mini-hexapod stage.

### 3.2. The EDXAS_L branch   

The EDXAS_L branch serves a large variety of users ranging from chemists to materials scientists to solid state physicists. This branch hosts a new combined XAS/DRIFTS facility for structure/function studies of catalysts, and facilities for XAS/XMCD/XMLD investigations of magnetic materials at ambient conditions or high pressure and/or low temperature, using pulsed high magnetic fields, electromagnets or permanent magnet devices. Customized user experiments are also generally installed in this experimental hutch. An example is the facility for dynamic compression with high-power lasers.

#### 3.2.1. The combined XAS/DRIFTS facility   

**Figure 26:**
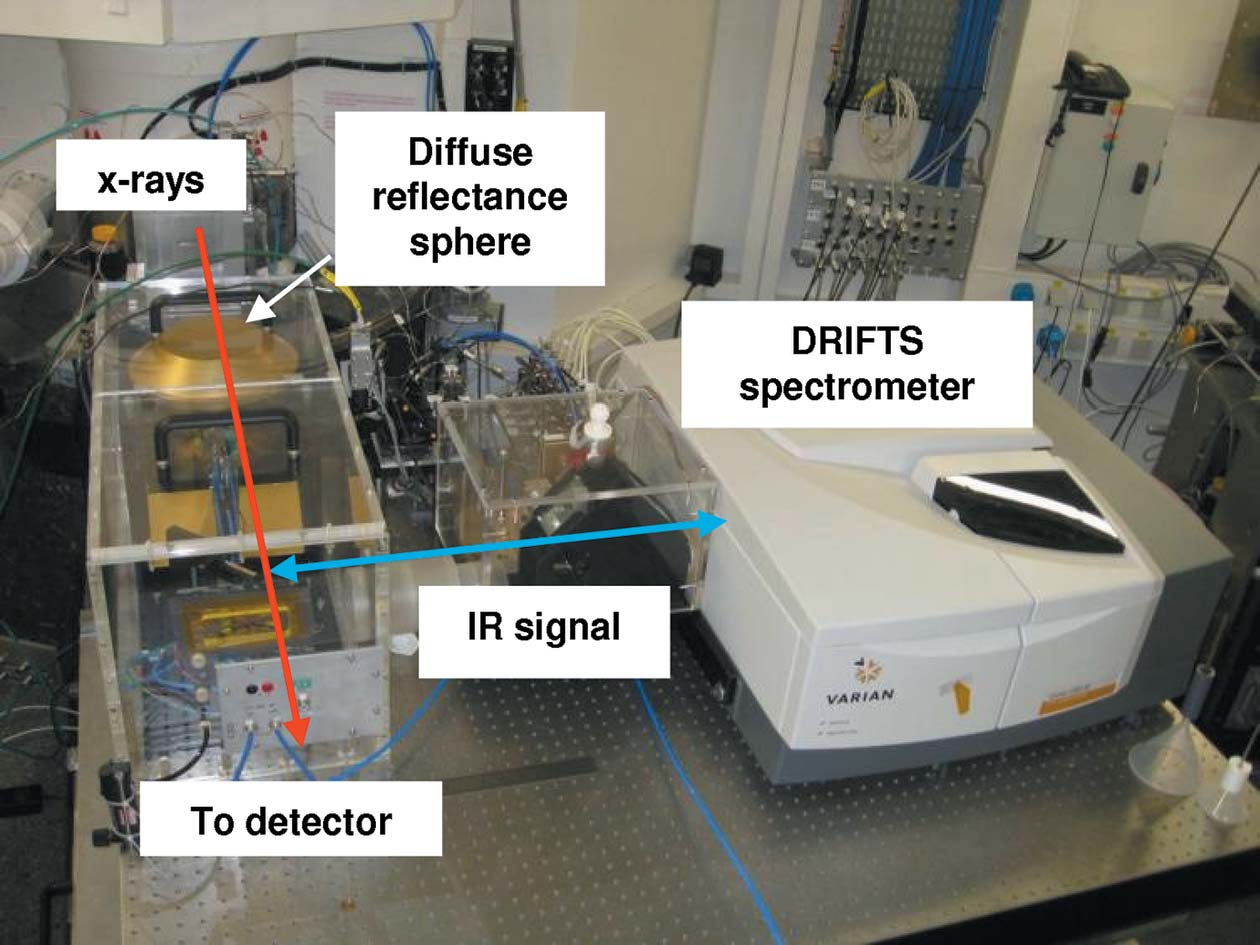
Photograph of the XAS/DRIFT/MS facility in EDXAS_L.

The EDXAS_L branch permanently hosts the DRIFTS spectrometer, used in synchronization with time-resolved EXAFS for *in situ* characterization of structure–function relationships in heterogeneous catalysts and other functional materials. The system is based on a Varian 680 FTIR instrument, a diffuse reflectance sphere, and a XAS/DRIFT/MS cell developed at the ESRF. The cell is positioned at the focal point of the diffuse reflectance sphere, and fine alignment is achievable by a vertical movement of the whole cell and by three motorized motions of the spherical mirror. The whole setup is mounted inside a Plexiglas box with N_2_ flux to minimize H_2_O and CO_2_ IR signals. A photograph of the setup mounted on EDXAS_L is reported in Fig. 26 ▸.

The cell is a plug flow reactor for powder samples in an interchangeable crucible to adjust sample thickness for absorption measurements. A small dead volume (about 0.5 cm^3^) allows kinetic studies to be performed. The operational temperature range is 300–970 K. To minimize the temperature gradient along the catalytic bed, the incoming gas feed is pre-heated as it flows through the heater before interaction with the sample. The body of the cell is made of an Inconel alloy for its resistance to reducing and oxidizing atmospheres even at high temperature. The IR windows are made of ZnSe, transparent in the mid-infrared, while vitreous carbon windows were chosen for the X-ray beam. Both materials have a melting point higher than 1500 K and are suitable for use in catalysis experiments.

#### 3.2.2. XAS/XMCD facilities for studies of magnetism at high pressure or magnetic field   

Pressure is an effective variable for studying the complex interplay between magnetic and structural degrees of freedom. Because pressure acts directly on interatomic distances, band hybridization strength can be tuned which may induce magnetic instabilities leading to structural phase transitions, to non-ferromagnetic phases or to partial suppression of magnetic moments or ferromagnetic order. *K*-edge X-ray magnetic circular dichroism (XMCD) is a powerful probe to investigate the effect of pressure on structure and magnetism in 3*d* metals and their compounds (Mathon *et al.*, 2004 ▸; Torchio *et al.*, 2014*b* ▸). Along with temperature and pressure, high magnetic fields can also tune matter through various structural and magnetic phases featuring novel and exotic properties. Very high magnetic fields are sometimes required to reach these phases, and pulsed magnets offer an economic and flexible path to fields beyond those generated by superconducting and resistive magnets currently available at synchrotron sources (Mathon *et al.*, 2007 ▸; Sikora *et al.*, 2009 ▸; Strohm *et al.*, 2012 ▸). Due to the elemental and orbital selectivity, XAS and XMCD are particularly suited to probe these phases independent of their state of crystallinity. To perform these experiments, ID24 takes full advantage of its intrinsically parallel and fast acquisition scheme. Circularly polarized X-rays are produced by means of a thin diamond or silicon crystal (quarter-wave plate) positioned at the exit of the polychromator chamber. A compact goniometer for the quarter-wave plate is placed in the restricted space between the polychromator and MV2. The main constraint of the energy-dispersive geometry is that the different energies diffracted by the curved Si crystal must satisfy Bragg’s law simultaneously on the quarter-wave plate crystal. This can be achieved by adjusting the angle between the two diffraction planes (Pizzini *et al.*, 1998 ▸).

**Figure 27:**
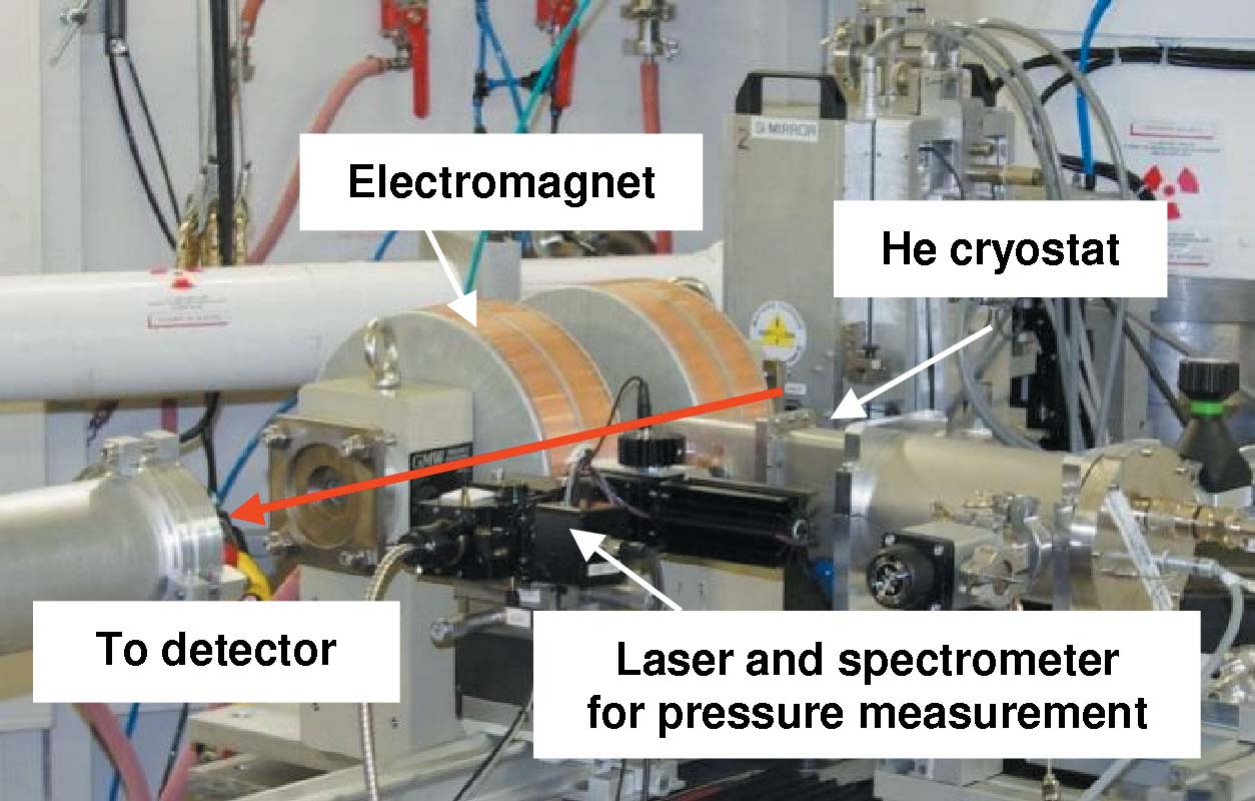
The XMCD facility for high-pressure and low-temperature studies.

Fig. 27 ▸ illustrates a photograph of the XMCD facility for high-pressure and low-temperatures studies installed in EDXAS_L. The facility features an electromagnet (0.75 T with 50 mm gap) and He cryostat (4–300 K) for the DAC. The pressure on the sample is measured using the fluorescence line of a small ruby chip placed close to the sample.

Different experimental facilities are available for pulsed magnetic field applications: (*a*) an ESRF-made minicoil (Van der Linden *et al.*, 2008 ▸) and (*b*) the LNCMI coil (Frings *et al.*, 2006 ▸), provided through a scientific collaboration with Laboratoire National Champs Magnétiques Intenses (LNCMI, Toulouse, France). The ESRF minicoil system with a stored energy of 4 kJ currently provides peak fields of 30 T for 50 µs at repetitions of up to 5 cycles min^−1^, in a sample space of 3 mm diameter with sample temperatures ranging from 4 to 250 K. The absolute uncertainty in field is 4% and pulse-to-pulse uncertainty is 0.1% at 30 T. Uncertainty in the timing is ∼2 µs. The LNCMI coil provides the same peak field but a much longer pulse (∼20 ms), thanks to the high-power generator (1 MJ). ID24 features a dedicated sample cryostat integrated within this facility, to reach temperatures down to 1.5 K in a diameter of 9 mm. This is sufficient to accommodate miniature high-pressure cells for future experiments under multi-extreme conditions.

#### 3.2.3. Facilities for customized user experiments: laser-driven dynamic compression   

**Figure 28:**
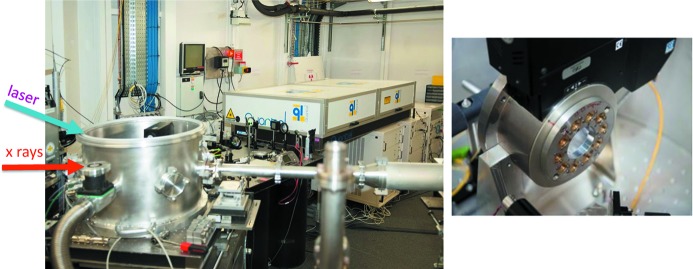
Left: photograph of the EDXAS_L experimental station hosting the Quantel laser from CEA (Bruyères-le-Châtel, France) and the target vacuum chamber during the dynamic compression experiment. Right: the wheel with the Fe targets.

Dynamic compression using high-power laser shock combined with X-ray techniques can be used to explore the atomic structure of matter in states that go beyond the limit of static compression. Recently XAS has been used to characterize the electronic and local structure in highly compressed SiO_2_ (Denoeud *et al.*, 2014 ▸) and Fe (Ping *et al.*, 2013 ▸) at large laser facilities. A first feasibility experiment to measure the Fe *K*-edge XAFS on a thin shocked foil on a synchrotron beamline was recently performed on ID24 (Torchio *et al.*, 2014*a* ▸). Fig. 28 ▸ (left panel) shows a photograph of the target chamber, the high power laser (∼40 J, 4 ns pulse length) from Quantel on loan from the Commissariat Energie Atomique (CEA, Bruyères-le-Châtel, France).

The target chamber contains a rotator with ten targets (right panel). 100 targets were prepared for this first experiment, consisting of 4 µm deposited Fe films sandwiched between diamonds. The role of the diamonds was to confine the sample shock for a time interval of a few nanoseconds, to allow the 100 ps X-ray pulse to probe a thermodynamically stable state. The blue and red arrows indicate laser and X-ray beam propagation, respectively: the laser impinges at 30° with respect to the X-ray beam. The shock destroys the target, but these first feasibility tests demonstrated that good quality XAFS can be acquired using a single X-ray pulse.

### 3.3. Summary of main characteristics   

Table 2 ▸ summarizes the main characteristics of beamline ID24.

## 4. Example of results   

In this section we illustrate examples of recent experiments from each branch. Among the large variety of possible applications, we have chosen two that best illustrate the new capabilities of ID24.

### 4.1. *In situ* EXAFS of melts at *P*,*T* conditions relevant to Earth’s interior   

To reach earth’s inner core conditions (*P* > 300 GPa and *T* ≃ 5000 K) matter can be statically compressed in a DAC and laser heated ‘*in situ*’, or dynamically compressed using high-power laser shocks (see §4.2.2). In both cases the timescales for acquisition need to be short. For laser-shocked matter, acquisition times need to be below the nanosecond. Acquisition times need to be short also when ‘*in situ*’ laser heating samples in the DAC; recent studies have shown that molten matter under continuous laser heating is unstable because chemical reactions or decompositions occur at extreme conditions which alter the chemical state and speciation of the absorber. Laser heating also often induces sample geometry variations. Pulsed heating (where pulse duration is typically of the order of 10 µs to 100 ms) has been shown to help mitigate these drawbacks.

Moreover, a very small (a few µm full size) and stable focal spot is required, because samples and laser spots are small (∼20 µm for *P* > 100 GPa), and because only the central part of the laser spot is probed to reduce the influence of thermal gradients. The relative position between the sample, the laser spot and the X-ray beam must remain stable to within ∼1 µm. Pressure and temperature gradients as well as chemical reactions, if any, across the laser-heated spot need to be quantified using two-dimensional hyperspectral mapping methods, where a complete XAS spectrum is included in each pixel of the map. EDXAS is ideally suited to attack these challenging studies, because acquisition speed and stability in focal spot and energy scale are not linked.

**Figure 29:**
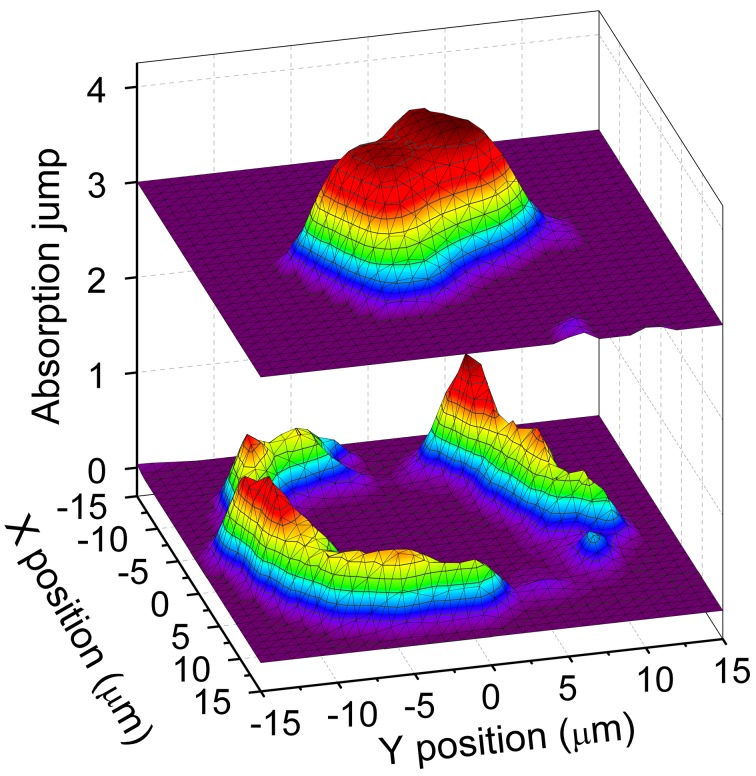
Ni *K*-edge jump map before (top) and after (bottom) laser heating of Ni foil. Total dimensions of the mapped area are 35 µm × 35 µm.

Fig. 29 ▸ illustrates the effect of strong spatial variations of temperature on a thin Ni foil in the DAC by mapping the Ni *K*-edge jump before and after laser heating. If the sample is not efficiently confined by a suitable pressure-transmitting medium, it can be destroyed upon melting.

**Figure 30:**
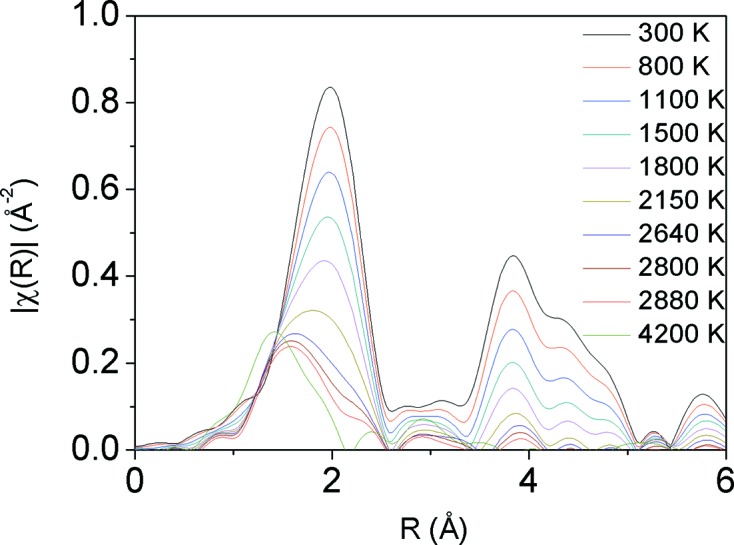
Evolution of the magnitude of Fourier transforms of Ni *K*-edge EXAFS during *in situ* laser heating of pure Ni foil to above the melting temperature, at 66 GPa. The most intense spectrum corresponds to Ni at ambient *T*. As *T* increases, the intensity of the peaks decreases and the first shell becomes asymmetric.

Very little can be found on the structure of liquid 3*d*, 4*d* or 5*d* metals, although much attention is being devoted to the calculation or measurement of melting curves in these systems (Anzellini *et al.*, 2013 ▸; Aquilanti *et al.*, 2015 ▸). Due to strong directional bonding arising from an incomplete *d*-electron valence band, atoms are not ‘spherical’ and compression of liquid *d*-metals could produce interesting non-uniform effects on the local structure. Together with Fe, Ni is an important alloying constituent of Earth’s interior: it is expected to be found both in the inner (solid) and the outer (liquid) cores. Therefore, besides fundamental interest, knowledge on local bonding properties in such systems at the relevant *P*, *T* conditions would have significant geophysical implications. Pure Ni has been compressed in a DAC and subsequently laser heated up to above melting (Kantor *et al.*, 2016 ▸). Fig. 30 ▸ reports the magnitude of Fourier transforms for a series of EXAFS at 66 GPa, from ambient temperature to ∼4200 K. Each spectrum results from ten accumulations of 100 ms exposures. The most intense spectrum corresponds to Ni at ambient *T*. As temperature increases, thermal vibrations damp both the first- and second-shell signal. At high temperature the first peak becomes asymmetric, while it continuously shifts towards lower distances. The onset of melting occurs when the second peak disappears while the first peak abruptly shifts towards smaller distances.

### 4.2. Towards XAS characterization of the warm dense matter regime   

Studying super-heated solids or strongly coupled plasma is critical to model many phenomena found in astrophysics, inertial confinement fusion, and applied processes of laser machining and ablation. These thermodynamical states are referred to as warm dense matter (WDM) due to the *P, T* ranges involved. Here, kinetic and potential energies are similar, standard material models break down, and there is no current capability to accurately predict the behaviour of matter under such conditions. Measuring the microscopic and atomic properties in the WDM regime is a challenging task. Recently, remarkable progress has been achieved using lasers to dynamically compress and heat the system and plasma-generated X-ray pulses (Denoeud *et al.*, 2014 ▸; Ping *et al.*, 2013 ▸) or X-ray free-electron lasers (Harmand *et al.*, 2015 ▸) to probe such extreme states. One of the future aims of the ESRF is to provide the dynamic compression user community the possibility to produce and probe samples in the WDM regime, with sufficient data quality allowing to validate theoretical models. In strong collaboration with external user groups, we are presently engaged in two directions to generate matter in the warm dense state at local thermal equilibrium: ohmic ramp heating and laser shock.

#### 4.2.1. Ohmic ramp heating   

In the first case, a thin (few micrometers) conducting sample is confined between two diamond windows and is rapidly heated along a quasi-isochoric path to several thousands of degrees by application of a fast current pulse. Due to thermal gradients across the windows, the cell explodes within a few milliseconds. The use of fast (200 µs) current ramps has shown that this method has the potential to reach *P, T* conditions in the 50 GPa and 10000 K range, bringing the metal well into the warm dense regime, before explosion of the cell.

**Figure 31:**
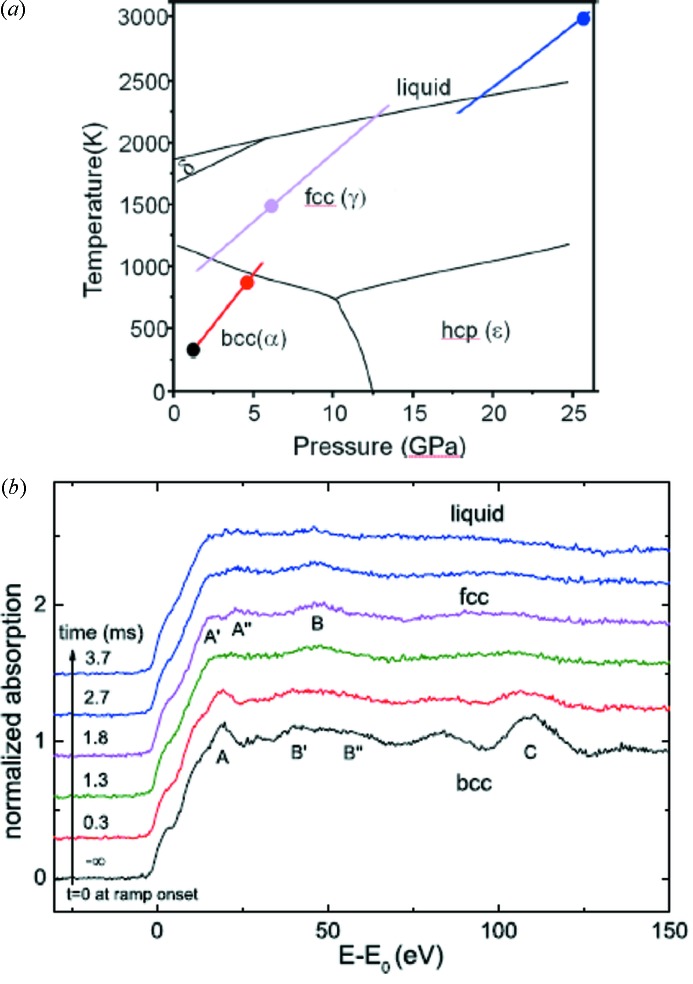
(*a*) Iron phase diagram and isochoric path for the bcc, fcc and liquid phases. The dots indicate roughly estimated *P, T* conditions for the black, red, pink and blue spectra shown in (*b*). (*b*) Sequence of Fe* K*-edge XANES selected during a fast ohmic ramp. The bottom spectrum (black) has been collected before the start. Acquisition time is 25 µs per spectrum. [Reprinted with permission from Marini *et al.* (2014 ▸). Copyright 2014, AIP Publishing LLC.]

We have performed on ID24 a first experiment using a relatively slow (20 ms) current ramp. A 5 µm-thick Fe foil was rapidly heated while recording a sequence of Fe *K*-edge XANES with 25 µs acquisition time and 50 µs repetition rate (Fig. 31 ▸). At the time of the explosion, the temperature of the sample was estimated to be close to 3000 K (Marini *et al.*, 2014 ▸). The WDM regime was not reached because ramp heating was too slow to compete with thermal expansion, and the cell blew up before the sample temperature could reach the interesting temperature regime. The spectra shown in Fig. 31(*b*) ▸ show that, within a few milliseconds, two structural phase transitions are observed, which transform the ambient body-centered cubic (bcc) phase of Fe into the face-centered cubic (fcc) phase, and then into the liquid phase (see the phase diagram in Fig. 31*a* ▸). The quality of this single-shot sequence was sufficient to probe the local atomic and electronic structure of the system at many steps along the ramp and to compare with *ab initio* full multiple scattering simulations of the solid and liquid phases. In the future this experiment can be repeated on faster timescales with the XH detector. The latter enables a sequence of XANES data to be recorded with a repetition rate of a few microseconds and the use of a faster current ramp (200 µs) enables the sample to reach temperatures of the order of 10000 K in a timescale that remains well below thermal expansion.

#### 4.2.2. Laser shock compression   

In this example, we exploited the time structure of synchrotron radiation, and synchronized a single X-ray bunch with a high-power laser pulse. The natural time structure of synchrotron X-rays has been historically used to perform pump-and-probe time-resolved experiments using single X-ray bunches (Wulff *et al.*, 2002 ▸; Lima *et al.*, 2011 ▸). In these studies, a sufficient signal-to-noise ratio is achieved by averaging multiple acquisitions of the same sample state and thus only repeatable phenomena are generally studied. Recently, due to improvements in source, optics and detectors, the possibility to obtain sufficient data quality using a single and unique 100 ps X-ray bunch was achieved using imaging (Rack *et al.*, 2014 ▸), diffraction and XAS techniques (Torchio *et al.*, 2014*a* ▸). In particular, the XAS results were achieved thanks to the very short integration time of the Ge XH detector (down to ∼80 ns), which allows only one bunch in the four-bunch filling mode of the ESRF storage ring to be selected.

**Figure 32:**
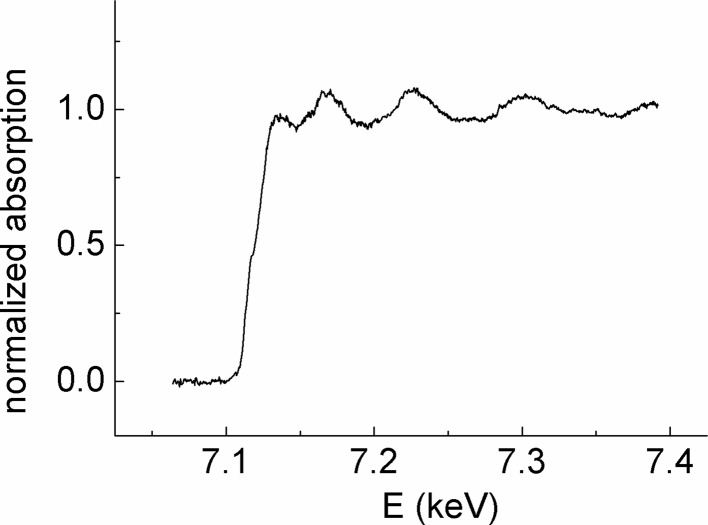
Fe *K*-edge XANES using a unique 100 ps bunch on an Fe target dynamically compressed by a laser shock. Pressure and temperature, estimated by hydrodynamical codes, are 100 GPa and 1400 K, respectively.

First Fe *K*-edge EXAFS measurements on a dynamically compressed Fe target performed on a synchrotron beamline using a unique X-ray pulse synchronized with a high-power laser pulse were recently achieved on ID24 (Torchio *et al.*, 2014*a* ▸). The small focused beam allows the use of a focused laser beam to generate the dynamical compression. This enables sample volume to be reduced and high energy density to be reached with relatively modest laser power. Using a portable 40 J laser, pressures and temperatures of 500 GPa and 13000 K were achieved, as estimated by hydrodynamic simulations. The shock lifetime in the iron target was confined for a few nanoseconds using a pair of diamond windows, providing a time window sufficiently large and homogeneous to probe the shocked sample with the 100 ps long X-ray pulse. Solid–solid and solid–liquid phase transitions of iron under extreme pressure and temperature were probed (Torchio *et al.*, 2015 ▸). Fig. 32 ▸ shows an example of Fe *K*-edge XANES measured on a dynamically compressed Fe target, at pressure and temperature of 100 GPa and 1400 K, respectively. This first experiment demonstrates the possibility to record good quality XAS data on dynamically compressed samples at a synchrotron beamline, and opens many exciting opportunities for probing the local and electronic structure in very dense states of matter.

## 5. Conclusion   

We present here the upgraded energy-dispersive XAS beamline ID24 at the ESRF. The new facility aims at covering the needs of the X-ray absorption spectroscopy users’ community in diverse scientific areas, from chemistry to catalysis, and from magnetism to the geosciences. It is particularly focused on time-resolved and extreme-conditions applications. The upgrade effort covered all instrumental areas: an optimized optical scheme, higher beam stability, faster and more efficient detection, new sample environments.

Conventional applications of the EDXAS spectrometer, such as probing in real time the electronic and local structure of a selected chemical species in a system undergoing a dynamical process, are extended to a wider time-resolution domain. We also show through several examples that the coupling of the EDXAS spectrometer to a high-brilliance source makes this facility very attractive for probing matter at extreme thermodynamical conditions that can be maintained for very short time. A few illustrative examples are shown of the new capabilities in studies of matter at pressures and temperatures relevant to Earth’s interior and beyond.

## Figures and Tables

**Figure 18:**
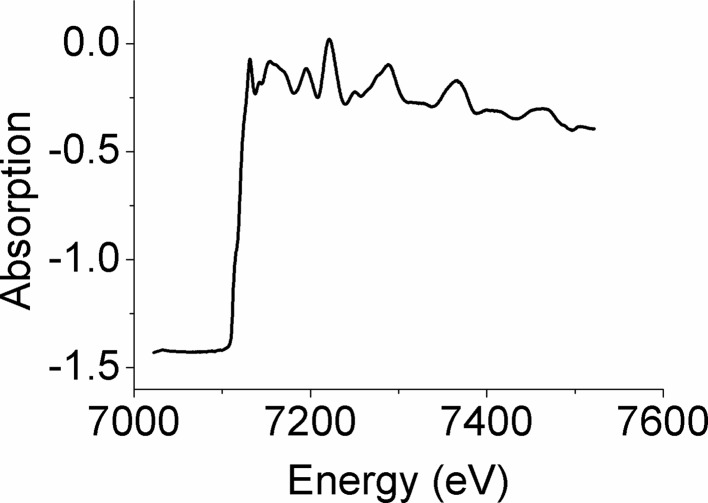
Example of Fe *K*-edge EXAFS of a standard 4 µm-thick polycrystalline Fe foil placed in a diamond anvil cell. The dimensions of the focal spot are those illustrated in Fig. 17 ▸.

**Figure 22:**
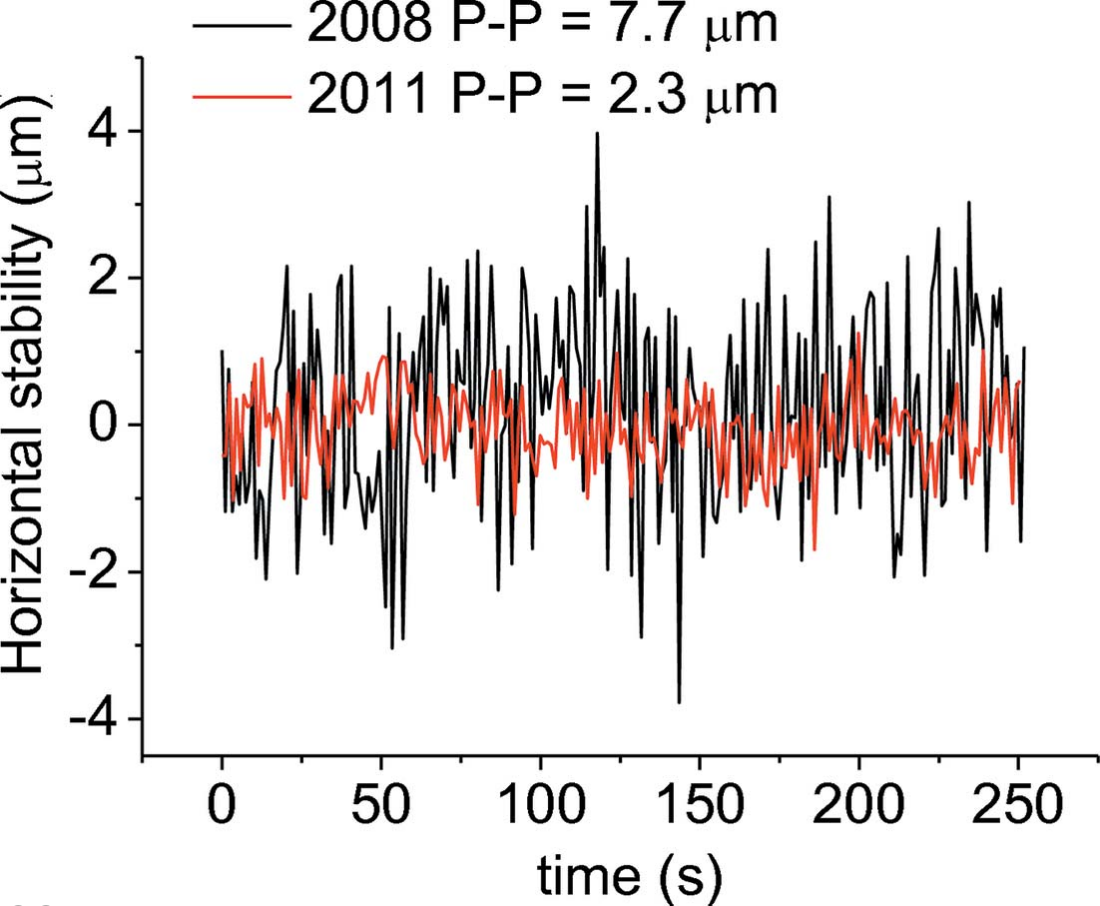
Movements of the beam in the horizontal plane, measured on the focal spot, as a function of time. A factor three reduction in PP amplitude is achieved with the new supports.

**Table 1:** Values of 2015 emittance, electron beam dimensions and divergence and size of the X-ray beam at 30 m from source (r.m.s. values); the latter are calculated for *E* = 12.4 keV and considering a total undulator length of 3.2 m

H	Emittance	nm	4
	Electron beam size	µm	409
	Electron beam divergence	µrad	11.9
	X-ray beam size at 30 m; 3.2 m ID; *E* = 12.4 keV	µm	509
V	Emittance	pm	5
	Electron beam size	µm	5.6
	Electron beam divergence	µrad	6.1
	X-ray beam size at 30 m; 3.2 m ID; *E* = 12.4 keV	µm	124

**Table 2:** ID24 beamline main characteristics

Beamline name	ID24
Source	U27 1.6 m + U27 1.4 m + Revolver U27/U32 1.4 m + U32 1.6 m
Primary slits	3 mm × 1.5 mm located at 26.8 m
Polychromator	Si(111) or Si(311)
Mirrors	Two mirrors in KB configuration @ 3 mrad (Pt/Si/Rh coating)
Energy range	5–28 keV
Beam size at 7 keV (FWHM)	3 µm × 3 µm using a Si (111) Bragg polychromator and a vertically refocusing Si mirror at 4.5 mrad
Flux on sample at 7 keV	1 × 10^14^ photons s^−1^ at 200 mA with Si(111) Bragg polychromator and two Si KB mirrors at 3 mrad†
Beam size at 24 keV (FWHM)	30 µm × 100 µm (FWHM) using a Si(111) Laue polychromator and no vertically refocusing mirror
Flux on sample at 24 keV	4 × 10^13^ photons s^−1^ at 200 mA with Si(111) Laue polychromator and two Pt-coated KB mirrors at 3 mrad
Detectors	Two-dimensional FReLoN CCD camera, one-dimensional Hamamatsu CCD camera, XH Ge microstrip detector
Sample environments	*In situ* laser heating for the DAC (*P* < 300 GPa, *T* < 5000 K), He-flow cryostat for DAC, high-temperature reactors, plug flow capillary microreactors, DRIFTS spectrometer, stopped-flow cell, UV–Vis spectrometer, pulsed magnetic fields (*B* < 30 T) and various magnet devices for XMCD and XMLD

† Measured with a calibrated Si diode.
